# Automated Force Field Developer and Optimizer Platform: Torsion Reparameterization

**DOI:** 10.1021/acs.jcim.6c00528

**Published:** 2026-03-09

**Authors:** Alejandro Blanco-Gonzalez, William Betancourt, Ryan Michael Snyder, Shi Zhang, Timothy J. Giese, Zeke A. Piskulich, Andreas W. Götz, Kenneth M. Merz, Darrin M. York, Hasan Metin Aktulga, Madushanka Manathunga

**Affiliations:** † ATTMOS Inc., 325 E Grand River Ave Ste 328, East Lansing, Michigan 48823, United States; ‡ Department of Computer Science and Engineering, 3078 Michigan State University, East Lansing, Michigan 48824-1322, United States; § Laboratory for Biomolecular Simulation Research, Center for Integrative Proteomics Research and Department of Chemistry and Chemical Biology, 242612 Rutgers University, Piscataway, New Jersey 08854-8087, United States; ∥ San Diego Supercomputer Center, 8784 University of California San Diego, La Jolla, California 92093-0505, United States; ⊥ Department of Chemistry and Department of Biochemistry and Molecular Biology, Michigan State University, East Lansing, Michigan 48824-1322, United States

## Abstract

General force fields such as General Amber Force Field (GAFF) have been designed for broad applicability and are widely used in protein–ligand binding simulations in structure-based drug discovery. However, the force field parameters are not always transferable across ligand molecules, and custom reparameterization is sometimes necessary for accurate binding free energy simulations. This is especially true for torsion parameters, which are highly dependent on stereoelectronic and steric effects. Here, we report a novel, flexible, and user-friendly computational tool called the Automated Force Field Developer and Optimizer (AFFDO) platform that allows generating accurate, tailored GAFF2 torsion parameters for drug-like molecules. For a given ligand, AFFDO selects the most important torsions, carries out GPU-accelerated density functional theory calculations to collect reference data and fits torsion terms using a fast gradient-based optimizer that leverages automated differentiation. We benchmark AFFDO by parametrizing a series of drug-like molecules and carrying out protein–ligand relative binding free energy (RBFE) simulations. The results show that AFFDO can significantly improve GAFF2 torsion parameters against QM reference data, which in some cases translates into better agreement with experimental RBFE values within a reasonable computational time.

## Graphical abstract

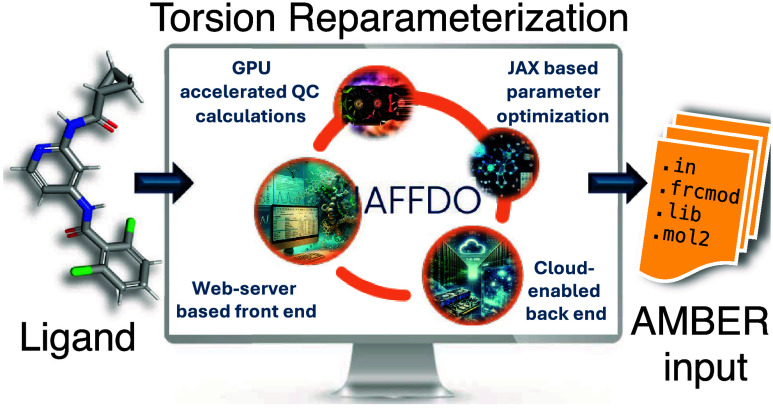

## 1 Introduction

Predicting binding free energy between a ligand and a target protein is an important task in structure-based drug discovery.^1,2^ This is usually done using computational methods such as free energy perturbation and thermodynamic integration, where molecular mechanics force fields serve as the main workhorse.^3^ The accuracy of the force field used is crucial for reliable predictions of free energy, thus for identifying promising drug candidates. Over the past few decades, several highly successful force fields such as AMBER,^4^ CHARMM,^5^ OPLS,^6^ and OpenFF^7,8^ have been developed for biochemical simulations. These force fields contain parameter sets for certain subsets of the chemical space. For example, in the widely used AMBER force field, ff19SB^9^ and OL21 ^10^ provide parameters for protein and DNA simulations, respectively. When a molecule falls outside of these subsets (e.g., an organic ligand), obtaining the necessary parameters can involve a complex, lengthy, and computationally demanding parametrization process.

Existing force fields provide a general parameter set (e.g., GAFF^11,12^ parameter set in case of AMBER) that covers a broad range of molecules by defining new atom types, adding more chemical groups and utilizing a heuristic pattern matching algorithm. However, a generic parameter set may fail to deliver accurate parameters, specifically for torsion angles and nonbonded interactions. Among bonded interaction terms, torsion parameters are particularly sensitive to the local environment of the target molecule, as various stereoelectronic and steric effects must also be considered in their optimization. Additionally, interactions such as resonance effects among aromatic rings can influence torsional profiles, even when modifications occur in nonlocal areas that may not be detected through chemical perception.^13^ These factors make torsion parameters less transferable compared to other valence parameters.

For reasons outlined above, torsion parameters are often subjected to reparameterization. A common approach to reparameterization is to start with a general force field and refine parameters for individual molecules, mainly by fitting against quantum chemical (QC) data. Multiple tools centered around this approach have been reported to date.^14–22^ Although torsion reparameterization based on QC data fitting can improve the accuracy of the free energy simulations, the process of fitting the parameters to numerous torsions is labor-intensive and may incur significant computational costs. One way to reduce computational costs is the use of machine learning methods such as the Accurate NeurAl networK engINe for Molecular Energies (ANI)^23,24^ and modern semiempirical methods like xtb,^25^ which is a compromise between the accuracy and the computational cost to compute the reference data. Parameterize,^26^ a tool that allows the reparameterization of GAFF2/OpenFF torsion parameters, is a good example in this context.

In this paper, we report Automated Force Field Developer and Optimizer (AFFDO), a new GPU-accelerated software platform for force field parametrization. The current version of AFFDO is available at https://www.attmosdiscovery.com/tools as a web service free of charge. This free version allows reparameterization of GAFF torsion terms for molecules containing up to 90 atoms.

AFFDO optimizes the parameters in a force field by first generating high-quality QC data and then fitting against these data through a highly optimized end-to-end workflow; the implementation reported in this manuscript is specific to AMBER and targets torsion parameters only. However, extending AFFDO to generate parameter files compatible with other MD simulation packages such as GROMACS is planned for future development. Computational optimizations in AFFDO span multiple steps of the workflow. First, for a given ligand, significant torsions are identified by sampling the QC potential energy surface (PES); torsional scans are performed only for these significant torsions, eliminating unnecessary costs. Second, torsional scans are carried out using a low-cost semiempirical (xtb) method and are later refined using massively parallel, GPU-accelerated QC calculations. Finally, optimizations of force field parameters against QC scans are performed using a fast gradient-based optimizer that leverages automated differentiation for rapid convergence.

Using the AFFDO platform, custom torsion parameter optimizations can be completed for target molecules in a relatively short time (compared to the time it takes to carry out the corresponding free energy computations) and without user intervention. AFFDO allows step-by-step monitoring of the process and modifications by the user can be applied to any step of the workflow. We demonstrate the utility, accuracy, and performance of AFFDO by generating torsion parameters for a series of drug molecules. We then show how the reparameterized force field performs in predicting the relative binding free energy (RBFE) values of several protein–ligand systems reported in a widely used public data set.^27^ The next sections of this paper are organized as follows. In Section 2, we provide methodological details and describe the implementation of the AFFDO platform. Section 3 is devoted to results and discussion where we present the results of benchmark simulations, including timing data. Finally, we conclude this study and provide future directions in Section 4.

## 2 AFFDO Workflow

For a set of *N* atoms described by their coordinates in 3D Cartesian space $R^{3\times N}$ , the functional form for the AMBER force field, which is largely shared by CHARMM and OPLS force fields as well, is given by eq 1.

1 $$V\left(R^{3\times N}\right)=\sum_{i\in \mathrm{bonds}}k_{b_{i}}{\left(l_{i}-l_{i}^{0}\right)}^{2}+\sum_{i\in \mathrm{angles}}k_{a_{i}}{\left(\theta_{i}-\theta_{i}^{0}\right)}^{2}+\sum_{i\in \mathrm{torsions}}\sum_{n}\frac{1}{2}V_{i}^{n}\left[1+\cos\left(n\omega_{i}-\gamma_{i}^{n}\right)\right]+\sum_{i<j}\left[s_{\mathrm{ij}}^{\mathrm{LJ}}\varepsilon_{\mathrm{ij}}\left({\left(\frac{r_{\min,\mathrm{ij}}}{r_{\mathrm{ij}}}\right)}^{12}-2{\left(\frac{r_{\min,\mathrm{ij}}}{r_{\mathrm{ij}}}\right)}^{6}\right)+s_{\mathrm{ij}}^{C}\frac{q_{i}q_{j}}{4\pi \varepsilon_{0}r_{\mathrm{ij}}}\right]$$

Here, the bond (first term) and angle (second term) energies are approximated as harmonic oscillators, where *l*_*i*_^0^ and θ_*i*_^0^ denote the equilibrium bond length and bond angle, respectively, and *k*_*b*_*i*__ and *k*_*a*_*i*__ are the corresponding force constants. The torsional contribution (third term) models the energy associated with rotation about a bond and is expressed as a Fourier series to account for multiple periodic contributions arising from chemical effects such as bond order, conjugation, and neighboring substituents. *V*_*i*_^*n*^ is the barrier height of the *n*-th Fourier component, ω_*i*_ is the dihedral angle, *n* is the periodicity, and γ_*i*_^*n*^ is the associated phase offset.

Nonbonded interactions (final term) describe short-range Pauli repulsion and van der Waals dispersion via a Lennard–Jones potential, together with electrostatic interactions between partial charges. The nonbonded sum runs over atom pairs *i* < *j* that are not excluded by molecular topology, following standard AMBER conventions (e.g., exclusion of 1–2 and 1–3 interactions). Interactions between atoms separated by three bonds (1–4 pairs) are included with topology-dependent scaling, using distinct scaling factors for the Lennard–Jones and electrostatic contributions (SCNB and SCEE, respectively), encoded in the pair-dependent scaling coefficients *s*_*ij*_^LJ^ and *s*_*ij*_^C^ appearing in eq 1, while all more distant pairs (1–5 and beyond) interact without additional scaling. Long-range electrostatic interactions are evaluated using particle mesh Ewald–type methods.^28^

Parameterization of a new molecule involves determining the optimal values for terms in eq 1. While generic force fields such as GAFF can serve as a good model for organic molecules and pharmaceutical compounds in general, it is unreasonable to expect high fidelity from a generic force field consistently, given the practically infinite size of the set of organic molecules and pharmaceutical compounds. Creating force field descriptions customized to a target molecule has been the main theme behind efforts such as OpenFF BespokeFit^14^ and OPLS3e,^29^ too. Much like OpenFF BespokeFit and OPLS3e, the current customization targets in AFFDO are the torsion parameters because they are crucial for accurate structural representation of molecules. Horton et al.^14^ describe the general methods and software tools used for customizing OpenFF’s torsion parameters; Roos et al.^29^ describe a significant expansion of the OPLS3e torsion angle database to improve the fidelity of OPLS along with a tool that can help users create their own custom OPLS torsion parameters.

As discussed above, QM-based torsion fitting is an established approach; accordingly, AFFDO’s contribution is not the concept of torsion fitting itself, but the specific integration and automation choices described below. AFFDO’s end-to-end workflow design emphasizes throughput and fully automatic operation without user involvement. In addition to this general motivation, AFFDO introduces several workflow-level developments that go beyond torsion fitting. First, AFFDO includes a torsion-focused fragmentation module (FragMentor) that operates as an initial, fully integrated step of the pipeline: it evaluates whether the molecule contains potentially reparameterizable torsions and uses these targets to decide whether QM scans should be performed on the full molecule or on automatically constructed fragments, only when this reduces QM cost without compromising the relevant local torsional environment. Second, AFFDO implements an automated procedure to identify and prioritize chemically significant torsions from conformer ensembles, avoiding unnecessary fitting. Third, torsion fitting is performed using a gradient-based optimizer that leverages gradient information (L-BFGS-B; SciPy/JAX implementation) to efficiently refine torsion parameters. In addition, several implementation-level advances were required to support high-throughput execution (with GPU acceleration where applicable) in practice, including automated torsion scans and constrained optimizations in the QM workflow (DL-Find + QUICK integration) and RESP-charge computation within QUICK. These implementation details are described in later sections.

For optimizing the torsion parameters for a molecule, it is necessary to determine the optimal barrier height, phase, and periodicity terms for each torsion in the molecule. The underlying torsion parametrization procedure of AFFDO is summarized in Figure 1. This multistep procedure achieves five main tasks: torsion-focused fragmentation (step 1), identification of significant torsions (step 2–7), generation of reference data (step 8), torsion fitting (step 9) and generation of the final output files (step 10).

**Figure 1:**
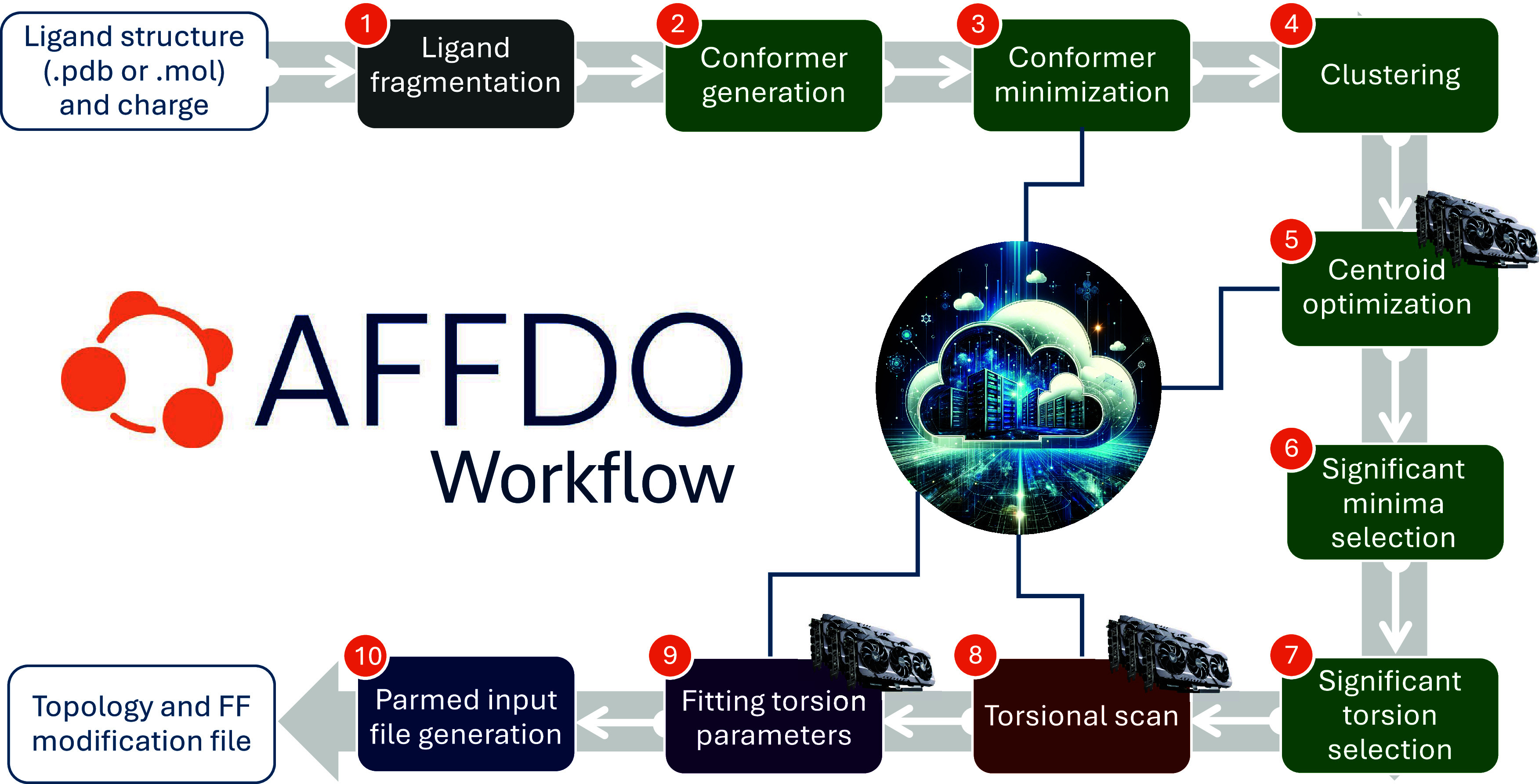
Parameterization procedure of AFFDO workflow. Steps 3, 5, 8, and 9 involve CPU or GPU parallelization and are carried out on a user-specified backend. Different tile colors gray, green, brown, violet, navy blue represent the five main tasks of the workflow: ligand fragmentation (step 1), identification of significant torsions (step 2–7), generation of reference data (step 8), torsion fitting (step 9), and the generation of final output files (step 10).

### 2.1 Ligand Fragmentation

The parametrization of large ligands can be challenging due to computational cost and potential steric clashes during torsional scans. This can be avoided by fragmenting the ligand, parametrizing the fragments separately, and transferring torsion parameters from fragments to the original ligand at the end of the procedure. AFFDO is equipped with a dedicated fragmentation tool, FragMentor, specifically designed for torsion parametrization. Its primary novelty lies in its fully automatic decision-making, which autonomously determines whether ligand fragmentation is necessary based on predefined criteria. This ensures that fragmentation is only applied when beneficial, aligning with AFFDO’s streamlined and automated workflow.

FragMentor strategically selects cleavage sites to target torsions suitable for parametrization while preserving their original chemical environments. The fragmentation decision and fragment sizes are guided by the number of *backbone-connected* torsions and by ensuring that torsions arising from distinct molecular regions are not forced into a single fragment, preventing excessive fragmentation while ensuring that chemically meaningful torsional regions are properly parametrized. Here, *backbone-connected* torsions refer to the subset of significant torsions whose central bonds lie on the longest non-hydrogen chain of the ligand (identified via a graph-theoretic longest-path filter before fragmentation). FragMentor operationally preserves the local chemical environment by partitioning significant torsions into connected *torsion regions* (consecutive torsions on the molecular graph) and generating a separate fragment for each region; fragments are retained only if they contain the atoms defining the region. Torsions within rings, end-of-chain torsions, and functional groups are explicitly excluded from cleavage, preserving key molecular substructures. The resulting fragments contain unique torsions, ensuring that the derived torsion profiles remain representative of the parent molecule. FragMentor also employs RDKit-based algorithms^30^ to break bonds and cap fragment ends with methyl groups. Additionally, detailed atom-index mappings between fragments and the original ligand (ligand-fragment atom maps) are systematically stored in JSON format, enabling accurate reconstruction and parameter transfer after optimization. Overall, FragMentor, integrated within AFFDO’s fully automated pipeline, reduces manual intervention and improves computational efficiency.

We note that related fragmentation tooling has been developed to reduce the cost of QM torsion drives, including OpenFF-Fragmenter.^13^ In its recommended WBO-sensitive mode, OpenFF-Fragmenter follows a bond-centric strategy in which fragments are grown for a *driven* rotatable bond until the Wiberg bond order (WBO) of that bond matches the parent molecule within a user-defined tolerance (i.e., one fragment is constructed per targeted torsion-drive bond). In contrast, FragMentor is implemented as the entry-point of AFFDO and is optimized for end-to-end GAFF torsion reparameterization: it operates on the *set of torsions selected by AFFDO* and targets workflow-level efficiency by minimizing the number of fragments/AFFDO jobs, while preserving the local chemical context required for fitting (as described above). We emphasize that both tools share the goal of reducing torsion-drive cost, but they operate at different granularity (bond-by-bond WBO-driven growth versus workflow-integrated torsion-region coverage) and are optimized for different refitting workflows.

### 2.2 Identification of Significant Torsions

An important step of the torsion parametrization is the identification of chemically important torsions of a given molecule. The goal here is to eliminate the parametrization of unnecessary torsions and reduce the computational cost. To achieve this task we developed the following novel methodology. For a given molecular structure, AFFDO generates a large number of conformers using the ETKDG method^31^ implemented in RDKit.^30^ Generated conformer structures are minimized using the GFN2-xTB method^32^ implemented in xTB.^25^ To filter redundant conformations and improve efficiency, minimized geometries are clustered using SciPy’s hierarchical clustering approach.^33,34^ The algorithm groups conformers based on geometric and energetic similarity, iteratively merging those with minimal Euclidean distances (RMSD and energy) to ensure a structurally diverse yet nonredundant selection. In our implementation, each conformer is represented by a two-feature vector consisting of its heavy-atom RMSD to the lowest-energy conformer and its relative energy (Δ*E*) from the conformer-minimization step. To combine these features in a single Euclidean distance without an ad hoc unit conversion, we L2-normalize each feature across the conformer set and apply Ward hierarchical clustering to the resulting normalized feature vectors (SciPy^33,34^ linkage, method = “ward”).

AFFDO then identifies the lowest-energy centroid and eliminates other centroids above a configurable energy window intended as a pragmatic screening criterion to retain low-energy conformers while discarding high-energy outliers (default Δ*E*_keep_ = 3.5 kcal/mol). If a higher-level reference is requested (e.g., DFT in this work), the retained centroid conformers are reoptimized at that level prior to torsion identification; otherwise, the xTB-minimized centroids from the prescreening stage are used directly for the torsion-dispersion analysis. Specifically, for each candidate torsion, AFFDO computes the wrapped dihedral angles across the retained centroid set and retains a torsion if the maximum centroid-to-centroid dihedral difference exceeds a configurable cutoff (default 30°). The resulting torsion list can contain rotations about methyl/terminal groups and multiple torsion definitions arising from the same central bond. Such torsions are pruned using chemically motivated heuristics. In particular, torsions involving terminal atoms (atoms with only one heavy-atom neighbor) are excluded by default because their large centroid-to-centroid angle differences often arise from quasi-free rotations that are not representative of the conformational changes targeted for reparameterization; this filter is configurable. Torsions entirely contained within rings are also excluded by default (independent of ring size) because many ring torsions are conformationally constrained and typically exhibit limited angle variation across the centroid ensemble; this choice may be revisited in future versions to better accommodate more flexible cyclic systems such as medium-sized rings (8–11-membered) and macrocycles (commonly ≥ 12-membered), where torsional reparameterization can be more impactful. When multiple torsion definitions arise from the same bond, AFFDO identifies the longest non-hydrogen chain of the molecule using a graph algorithm and prioritizes the most “backbone-like” torsion based on how deeply the terminal atoms lie within the heavy-atom framework.^26^ The pruned torsion list from this procedure results in the ultimate torsions (hereafter, *significant torsions*) which will be used in later stages.

### 2.3 Generating Reference Data

Starting from each centroid structure identified in the previous step, 360° torsional scans are carried out for each significant torsion using the GFN2-xTB method on a uniform dihedral grid with a default spacing of 20° between consecutive scan points (18 grid points over 360°). The grid spacing is a configurable AFFDO setting (default 20° in this work). This is achieved by using the CPU-parallel xtb program.^25^ In principle, one could use a single centroid structure (e.g., the structure corresponding to the most stable minimum) for all torsional scans. However, due to steric clashes, torsional scans initiated from certain structures can result in abrupt spikes or shifts in the energy profiles. Given the low computational cost of GFN2-xTB, we mitigate this issue by starting scans from all centroids and selecting the smoothest energy profile for each torsion. In AFFDO, this selection happens automatically by ranking the candidate scans using a composite score that (i) favors profiles that are well-described by a low-order Fourier representation and (ii) penalizes unphysical roughness in the energy curve, particularly discontinuous jumps between consecutive scan points and excessive high-frequency jaggedness/curvature. The highest-ranked profile is retained for refinement and subsequent fitting. Next, selected energy profiles are refined using density functional theory (DFT) employing the PBE0-D3BJ functional^35^ with the 6–31G* basis set for neutral and cationic molecules, and the 6–31+G* basis set for anionic molecules.^36,37^ The GPU-accelerated QUICK QC program^38–43^ is employed for these constrained optimization calculations. Note that the constraint is applied as an explicit *dihedral-angle constraint* on the scanned torsion (rather than Cartesian positional constraints on the four atoms) during the constrained optimization. To achieve this task, we enabled constrained optimization capability in QUICK during this work.

We then recompute each profile using GAFF2 as a preliminary step to refine force field parameters through comparison between the MM energy profiles and higher-level reference curves. This process is facilitated by a new implementation added to AmberTools during this work. Specifically, we interfaced the sander program with the DL-FIND software^44^ to allow for constrained geometry optimizations using several algorithms and coordinate systems. The interface is made by directly linking the DL-FIND library with the sander executable. This greatly improves the computational performance because it avoids the use of system calls to launch and reinitialize the sander program at each optimization step to obtain the energy and forces. The default setting for geometry optimization is the limited-memory Broyden-Fletcher-Goldfarb-Shanno (L-BFGS) algorithm^45^ and a hybrid delocalized coordinate (HDLC) system.^46^ The coordinate system is constructed using the residue definitions in the AMBER topology, which we use to partition the system into fragments for the HDLC representation. The interface, included in the release of AmberTools25, allows select bond length, bond angle, and torsion angle values to be constrained during optimization.

### 2.4 Torsion Fitting

As our current implementation is specific to AMBER, AFFDO constructs the initial force field for a given ligand using GAFF/GAFF2 parameters^11,12^ and AM1-BCC,^47,48^ ABCG2,^49^ and RESP^38,50,51^ atomic charges. Specifically, the Antechamber program^52^ in AmberTools^53^ is used to assign atom types and parameters for the target ligand. The charges are computed for all centroid structures, according to the model charge selection, and final atomic charges are obtained by a Boltzmann averaging procedure. Specifically, AFFDO computes Boltzmann weights from the relative centroid energies Δ*E*_*k*_ = *E*_*k*_ – *E*_min_ at *T* = 298 K, *w*_*k*_ ∝ exp­(−Δ*E*_*k*_/(*k*_B_ *T*)) (normalized over the retained centroids), and then forms the per-atom averaged charges as *q̅*_*i*_ = ∑_*k*_ *w*_*k*_ *q*_*i*_^(*k*)^. This is because most of these charge models are highly dependent on the target molecular conformations.

To fit the torsion parameters, we developed a novel methodology that uses a quasi-Newton optimization algorithm that only requires gradient information, specifically the L-BFGS-B method implemented in the SciPy package^33,34^ coupled with Google’s JAX library.^54^ At a high level, BFGS and similar Quasi-Newton methods minimize an objective function using an iterative approximation of the function’s Hessian, *B*. For a given iteration *k*, search direction *p*_*k*_, step size generated by line search α_*k*_, and corresponding parameter update *s*_*k*_, we have the following eq 2

2 $$\begin{array}{l} p_{k}=-B_{k}\Delta f\left(x_{k}\right)\\ x_{k+1}=x_{k}+\alpha_{k}p_{k}\;\mathrm{and}\;s_{k}=x_{k+1}-x_{k}\\ y_{k}=\Delta f\left(x_{k+1}\right)-\Delta f\left(x_{k}\right)\\ B_{k+1}=B_{k}+\frac{y_{k}y_{k}^{T}}{y_{k}^{T}s_{k}}-\frac{B_{k}s_{k}s_{k}^{T}B_{k}^{T}}{s_{k}^{T}B_{k}s_{k}} \end{array}$$

These methods are more robust than simple gradient descent but notably still require the gradient of the objective function Δ*f*(*x*_*k*_). In this case, we define our objective function as the sum of squared error between QC and GAFF torsional energy profiles. In the present implementation, the objective targets agreement of torsional energy profiles; adding geometric terms is a natural extension for future development. Both profiles are generated by performing constrained minimizations on a uniform 20° dihedral grid, where the *scanned torsion itself* is enforced via an AMBER dihedral-angle restraint at each target angle (force constant 500 kcal/mol/rad^2^ with a ± 0.5° tolerance band around the target). We find that existing GAFF2 parameters generally serve as good initial guesses and ensure rapid convergence. The main challenge then remains rapid evaluation of the gradients of the loss function. We perform the torsional scans and loss function evaluations using an implementation of the AMBER force field written with Google’s JAX library, which is a set of function transformations for Python that are an extension of NumPy. Through its support for automatic differentiation (i.e., Autograd), we can obtain the gradient of the objective function to machine precision with no additional implementation overhead. These function transformations also enable acceleration on GPUs for energy evaluations and constrained geometry optimizations.^54^ In the context of a method like L-BFGS-B, this means that we can avoid the imprecision and massive overhead of finite difference or complex step approximations of the gradient of energy profiles. In this work, the ligand-specific fitting strategy used throughout the QM-benchmark and RBFE simulations refines torsional parameters by adjusting the barrier height, phase, periodicity, and the 1–4 electrostatic and van der Waals scaling factors (scee and scnb). These scaling factors modulate Coulombic and dispersion interactions between atoms three bonds apart, helping reproduce the local electronic environment more realistically and preventing overestimation of nonbonded contributions. AFFDO also provides a streamlined torsion-only variant in which only the torsional barrier heights are optimized while all other terms, including scee and scnb, remain fixed to their standard GAFF values. This alternative mode retains the standard GAFF nonbonded parametrization and provides a robust option when such ligand-specific tuning is not desired.

### 2.5 Generating Output Files

In this task, AFFDO prepares a series of files that can be directly used as input to Production Free Energy Simulation Setup and Analysis Framework (ProFESSA)^55^ to run binding free energy simulations. ProFESSA is an automated, flexible, end-to-end workflow for performing alchemical free energy simulations using AMBER’s GPU-accelerated MD engine^56–59^ and is centered around the Drug Discovery Boost package,^60^ which provides enhanced sampling features and analysis tools.^61–63^ Since the parameter fitting described above is performed by running the fitting algorithm for each significant torsion separately, it results in multiple topology files, each containing a set of modifications corresponding to the optimized parameters. Furthermore, if the parametrization were performed using ligand fragments rather than the full ligand, one would end up with multiple topology files significantly different from each other. Therefore, it is necessary to merge all the changes and construct a final topology file for the full ligand, but such a topology file will be inconvenient to use in ProFESSA. This is because during the setup of free energy simulations, the ligands are embedded in condensed phases (solvent and protein environments), and new topology files are created. To circumvent this issue, AFFDO processes these topology files and generates an input file for the parmed program^64^ of AmberTools.^53^ Furthermore, force field library (.lib), force field modification (.frcmod), and a coordinate file that also contains atomic charges (.mol2) are also generated. If the parametrization was performed using ligand fragments, ligand-fragment atom maps are taken into consideration during the preparation of above files. The resulting files can then be used to setup binding free energy simulations. The parmed input file can be used to update the solvent–ligand and protein–ligand topology files with new torsion parameters. Analysis of free energy simulation results were performed using the FE-ToolKit package.^65^

## 3 Benchmarking Studies

We benchmarked AFFDO by reparameterizing torsions for ligands drawn from the widely used Wang et al. data set.^27^ Specifically, we considered ligand series associated with the TYK2 and MCL1 systems, which comprise closely related analogs differing by modest functional-group substitutions, thereby mimicking a lead-optimization campaign.

Two benchmark components were evaluated. First, we assessed torsion-profile agreement by comparing GAFF2 versus AFFDO-optimized torsional potential energy surfaces against QC constrained dihedral scans. This torsion-profile benchmark includes 16 TYK2 ligands and 40 MCL1 ligands. Second, we evaluated the downstream impact of torsion reparameterization on RBFE predictions using the RBFE networks defined in the same data set; these RBFE results are reported for 15 TYK2 transformations and 14 MCL1 transformations (see Table S2 of the Supporting Information). We also report the timing and computational cost of torsion parameter optimization with AFFDO.

For these benchmarking tests, AFFDO was installed on a Linux server with Intel Core i7–12700K CPU (12 core) and 64 GB RAM. A cloud instance consisting of 4 NVIDIA RTX4090 GPUs, 36 AMD EPYC 7282 CPU cores and 64 GB memory was reserved from Vast.ai and configured as the backend of AFFDO. AmberTools/24, QUICK/24.03 and xTB/6.6.0 were installed on the cloud instance. For AmberTools and QUICK compilations, CUDA/12.0, GCC/9.3.0 and OpenMPI/4.0.3 were used.

### 3.1 Torsion Reparameterization Using AFFDO

Selected ligand structures were obtained from published data and used as input to the AFFDO workflow. Ligands were fragmented using FragMentor (Figure 2), and significant torsions were identified. The threshold energy value for conformer selection and the torsion angle difference between a pair of conformers for significant torsion identification were set to 3.5 kcal/mol and 30°, respectively. The resulting number of significant torsions for the representative ligands, jmc28 and L35, were 4 and 5, respectively.

**Figure 2:**
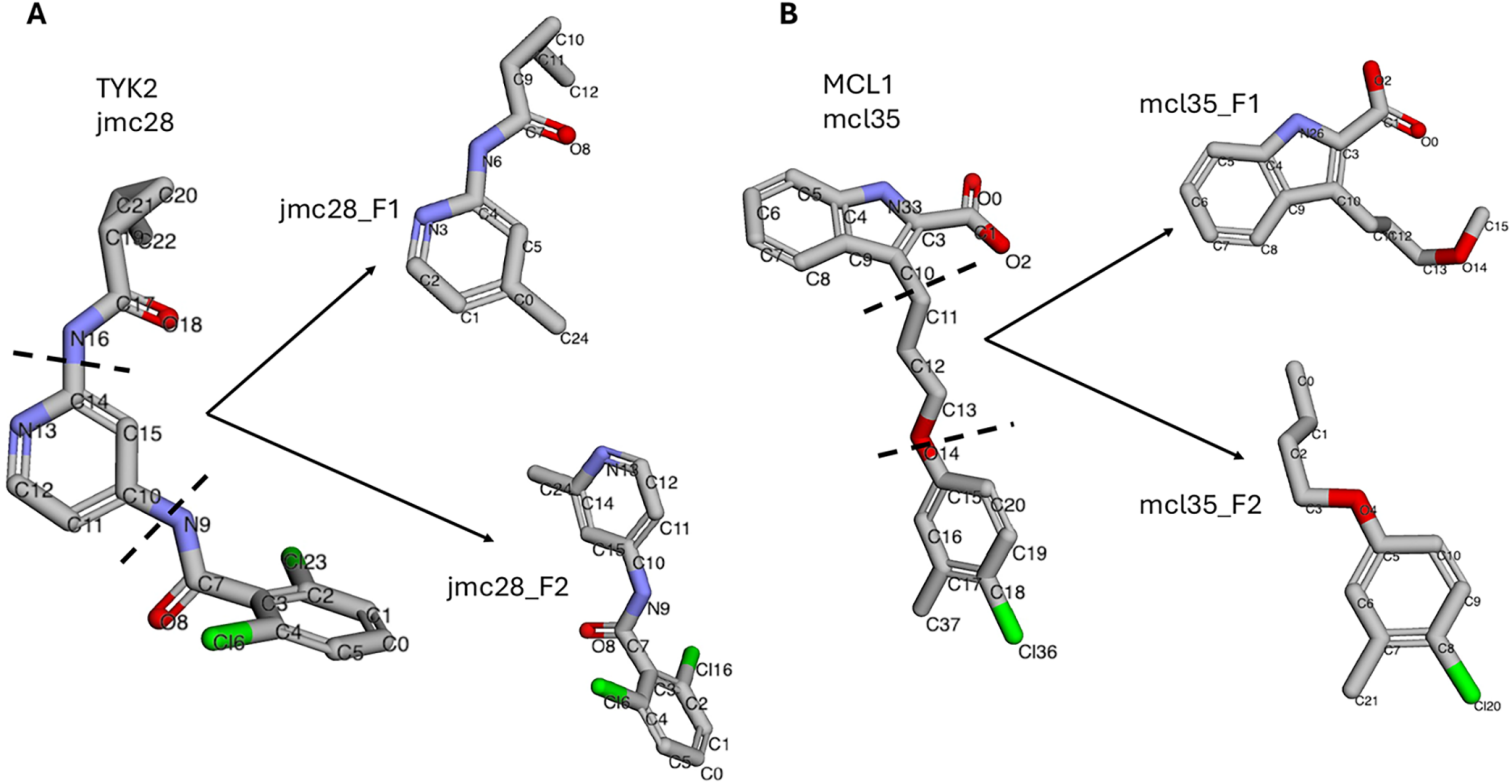
General fragmentation pattern of the representative ligands. Fragmentation of jmc28 ligand from TYK2 (A) and L35 ligand from MCL1 (B) protein systems are shown here. This AFFDO integrated feature streamlines the torsion parametrization process.

To generate the QC reference data, torsional scans of TYK2 ligands were carried out at the PBE0-D3BJ/6–31G* level of theory. For anionic MCL1 ligands, PBE0-D3BJ/6–31+G* level of theory was used. An interval of 20° was used for all scans. The default settings were used for parameter fitting (GAFF2 torsion parameters as initial guess, 0.05 step size, 0.001 kcal/mol RMSD convergence criteria). The torsional scan energy profiles were also computed using standard GAFF2, as well as using parameters optimized by AFFDO (“customized GAFF2”). The computed torsional scan energy profiles using QC, GAFF2 and AFFDO-optimized parameters of the most significant torsions are depicted in Figure 3. The plots for the remaining torsions are reported in Figure S1.

**Figure 3:**
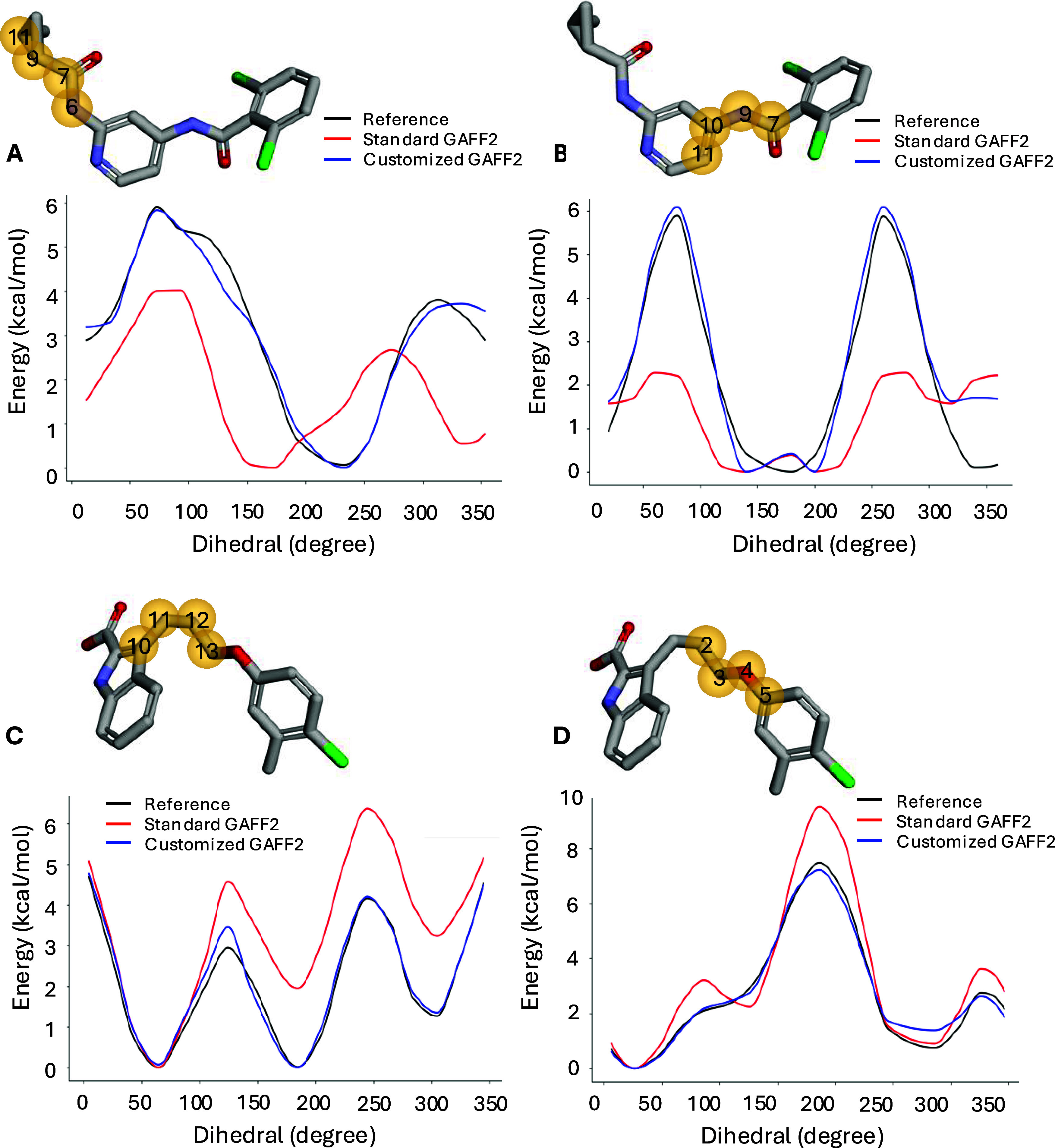
Torsional scan energy profiles of the most significant torsions of jmc28 ligand from TYK2 (A, B) and L35 ligand from MCL1 protein system (C, D). The corresponding torsions are highlighted in the molecular structures by yellow spheres.

As can be seen in Figure 3A,3B, standard GAFF2 poorly describes the selected torsions of the jmc28 ligand. Specifically, in the former (6–7–9–11 torsion), GAFF2 results in lower barrier heights and a phase shift with respect to the Reference QC energy profile. In the latter (7–9–10–11 torsion), the phase of GAFF2 is consistent with QC, but the barrier heights are significantly different and the minima are not correctly reproduced. The energy profiles of L35 (Figure 3C,D) suggest that GAFF2 describes the corresponding torsions properly. The minima are consistent with those of the QC PES although barrier heights are slightly different. As clearly evident from all cases, the reparameterization improves the torsional scan plots significantly, as customized GAFF2 correctly describes the selected torsions.

To quantitatively assess the improvements achieved by AFFDO, we computed root-mean-square error (RMSE), mean absolute error (MAE), and correlation metrics (Pearson and Spearman coefficients) for both TYK2 and MCL1 ligand parametrization. The results, presented in Table 1, compare GAFF2 and AFFDO performance relative to the QC reference data within each system. For clarity, these statistics quantify agreement with QC torsion-scan energy profiles evaluated over all dihedral-scan grid points and should not be interpreted as RBFE predictive accuracy. Metrics are computed from energy differences across the scanned dihedral angles using the QC (DFT) profile as reference. Additionally, RMSD values quantify deviations in optimized geometries relative to QC-optimized structures, providing further validation of the impact of torsion reparameterization on molecular conformations.

**Table 1:** Comparison of GAFF2 and AFFDO Torsion-Profile Accuracy for the TYK2 and MCL1 Ligand Sets^c^

	TYK2		MCL1	
Metric	GAFF2	AFFDO	GAFF2	AFFDO
MAE^a^ (kcal/mol)	1.56 ± 0.21	0.20 ± 0.06	0.81 ± 0.06	0.51 ± 0.06
RMSE^a^ (kcal/mol)	1.92 ± 0.29	0.24 ± 0.07	1.08 ± 0.07	0.69 ± 0.08
Pearson (*r*) (unitless)	0.57	0.96	0.90	0.94
Spearman (ρ) (unitless)	0.52	0.95	0.90	0.94
Max RMSD^b^ (Å)	0.44	0.39	0.63	0.63

a Uncertainties reported as 95% confidence interval, calculated analytically.

b RMSD values measure conformational deviations relative to QC-optimized structures.

c Metrics quantify agreement with QC torsional potential energy surfaces obtained from constrained dihedral scans evaluated on a 20° angular grid (i.e., scan-point energies) and should not be interpreted as RBFE predictive accuracy. The benchmark includes 16 TYK2 ligands and 40 MCL1 ligands.

AFFDO demonstrates substantial improvements in both systems, with the enhancements being particularly pronounced in TYK2. The RMSE reduction for TYK2 (1.92 → 0.24 kcal/mol) indicates a significant refinement in torsional parametrization accuracy, leading to a much closer match with QC energy profiles. Similarly, Pearson and Spearman correlation coefficients show a notable increase, confirming the improved consistency between AFFDO-optimized torsion potentials and the reference data. While the improvements in the MCL1 (1.08 → 0.69 kcal/mol) system are less pronounced, they remain consistent with the observed torsional energy profiles (Figures 3 and S1). In this case, the primary contribution of AFFDO lies in correcting the energy barrier heights rather than major phase shifts or minima realignments, which were already reasonably well captured by GAFF2. This analysis provides a direct measure of AFFDO’s effectiveness in refining ligand torsion potentials by demonstrating its capability to systematically reduce deviations from quantum mechanical reference data. These improvements highlight AFFDO’s ability to enhance force field accuracy, particularly in cases where standard GAFF2 struggles to correctly capture torsional energy landscapes.

### 3.2 Relative Binding Free Energy Simulations

To further investigate the practical implications of AFFDO-optimized parameters, we assess their impact on relative binding free energy (RBFE) simulations by evaluating the fidelity of the customized GAFF2 force field with reoptimized torsion parameters in a series of ligand transformations targeting TYK2 and MCL1 protein systems. The initial protein structures were obtained from published data^27^ and simulations were prepared using the ProFESSA workflow with the AMBER FF19SB force field for proteins, GAFF2 for ligands, and TIP4P-Ew^66^ for water molecules. A second set of simulations were prepared by updating the AMBER topology files with optimized torsion parameters obtained from AFFDO (using the torsion + scee/scnb fitting mode described above). All remaining topology terms, including the ligand partial charges (AM1-BCC as assigned by ProFESSA), were kept identical between the GAFF2 and AFFDO cases. Accordingly, any differences in RBFE performance reported here are attributable to the modified torsional terms rather than a change in the charge model. This was done by executing parmed with the AFFDO-generated input file and the corresponding aqueous- and complex-phase topology files. All simulations were executed on the aforementioned cloud instance with 4 RTX4090 GPUs. Three independent trials were performed for each RBFE edge; each trial used 21 λ windows with 2.4 ns equilibration and 5 ns production per window (see Section S3 in the Supporting Information for full RBFE protocol details).

In Figure 4, we report RBFE values computed using standard GAFF2 and reparameterized GAFF2 along with experimental data. Experimental and computed RBFEs for *all* transformations (standard GAFF2 and AFFDO-parametrized GAFF2), together with statistical uncertainties, are reported in Tables S2 and S3 (Supporting Information, Section S4). Three key insights emerge from these data. First, as shown in Figure 4A,B, torsion reparameterization improves the RBFE predictions of most ligand transformations. Notably, most points in blue (AFFDO) are closer to the dashed lines than the red points (GAFF2), indicating reduced deviation from experimental values. Across all transformations, including both improved and unchanged cases, AFFDO reduces the MAE of standard GAFF2 by approximately ∼0.4 kcal/mol. Among the subset of transformations (ca. 80%) where reparameterization is beneficial, the average reduction in absolute error is ∼0.5 kcal/mol, with the highest observed improvement reaching 2.45 kcal/mol (see Table S3 for this subset). Second, while it is reasonable to expect improvements in RBFE predictions for the TYK2 system due to substantial changes in torsional energy profiles after reparameterization (Figure 3A,3B), the consistent improvements observed for the MCL1 system were more surprising, given that the torsional energy barriers for its ligands were only moderately altered (Figure 3C,D). This suggests that even subtle modifications to the torsional landscape can enhance RBFE accuracy. Third, the uncertainty associated with RBFE estimates is consistently lower in reparameterized GAFF2, indicating improved convergence in free-energy calculations. For direct comparisons between standard GAFF2 and reparameterized GAFF2, uncertainties for both force fields are explicitly shown in Figure 4 (horizontal and vertical error bars, respectively), ensuring a statistically balanced comparison. These findings collectively reinforce the role of torsional refinement in enhancing force field performance, though its effectiveness varies across different ligand transformations due to factors beyond torsion parameters alone.

**Figure 4:**
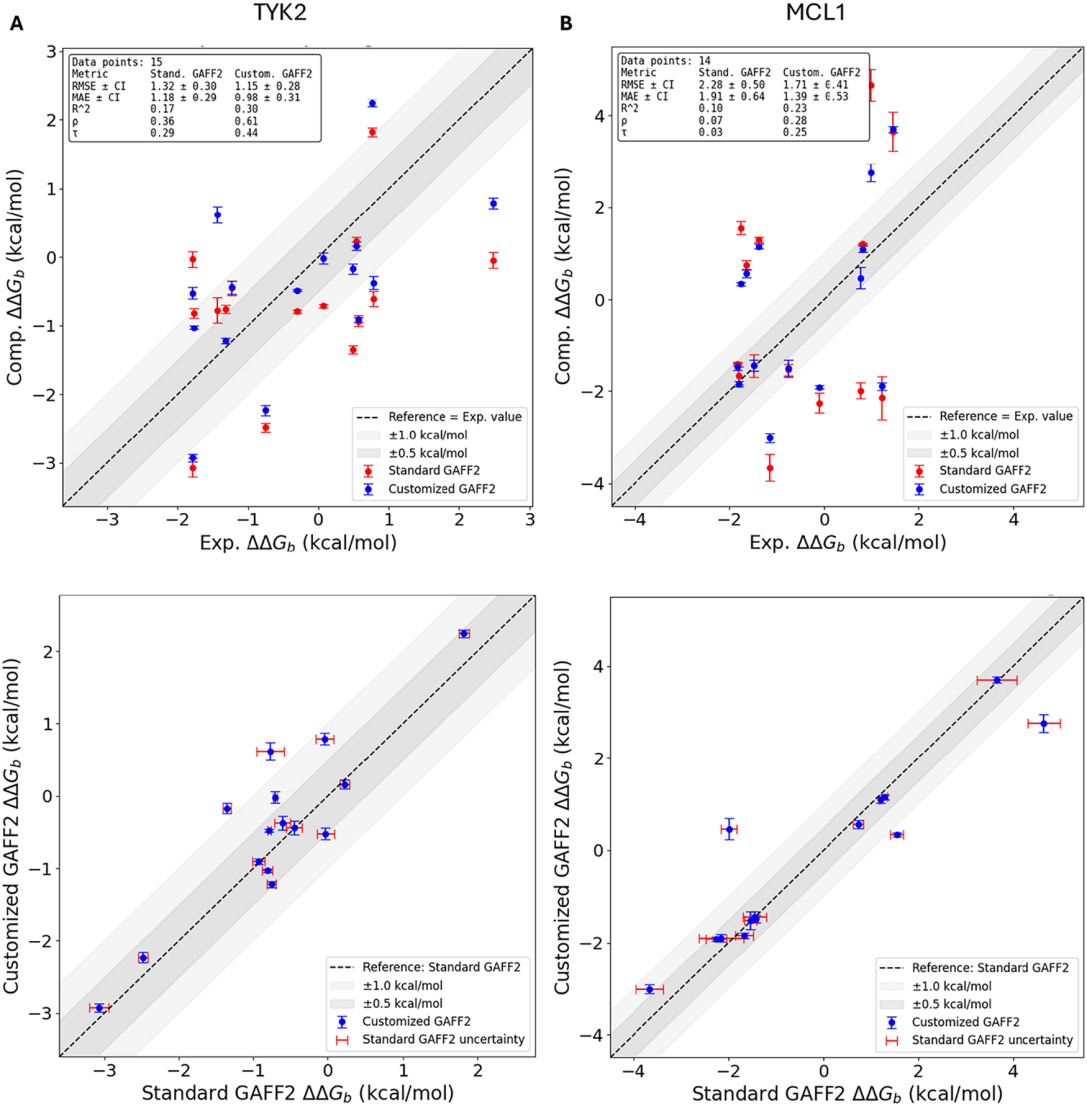
Computed versus experimental relative binding free energies (RBFEs) for the TYK2 (top left, panel A) and MCL1 (top right, panel B) ligand sets. Top panels compare experimental RBFEs to predictions from standard GAFF2 (red) and customized GAFF2 (blue). Bottom panels compare customized GAFF2 against standard GAFF2 predictions for each transformation. Error bars denote statistical uncertainty from three independent RBFE trials using identical analysis protocols (see Supporting Information); in the bottom panels, horizontal (red) and vertical (blue) error bars correspond to standard GAFF2 and customized GAFF2 uncertainties, respectively. The dashed line indicates perfect agreement. Insets report summary statistics for each force field (RMSE, MAE, *R* ^2^, Spearman ρ, Kendall τ, and *N*).

To elucidate how torsion reparameterization impacts RBFE accuracy, we analyzed the torsional sampling patterns from molecular dynamics trajectories of representative ligand transformations (see detailed analysis in Section S5 of the SI). We found that customized torsion parameters significantly redefined the ligand potential energy surfaces, influencing conformational accessibility across both end-states. For example, in the transformation of the TYK2 system jmc23-ejm55 (Figure 5), the repair of the torsion substantially increased the heights of the energy barrier and refined the minima regions, resulting in a narrower and more coherent conformational sampling between the ligands. This improved sampling overlap facilitated smoother alchemical transformations and directly enhanced the reliability of RBFE predictions compared to standard GAFF2. A similar effect was observed in the MCL1 system for the L49–L67 transformation (Figure S4 of SI). However, these improvements were not universal. For instance, in the ejm49–ejm50 (TYK2) (Figure S3 of SI) and L67–L27 (MCL1) (Figure S4 of SI) transformations, the optimized parameters did not substantially alter the conformational landscape sampled by standard GAFF2, resulting in RBFE estimates comparable to those obtained without reparameterization.

**Figure 5:**
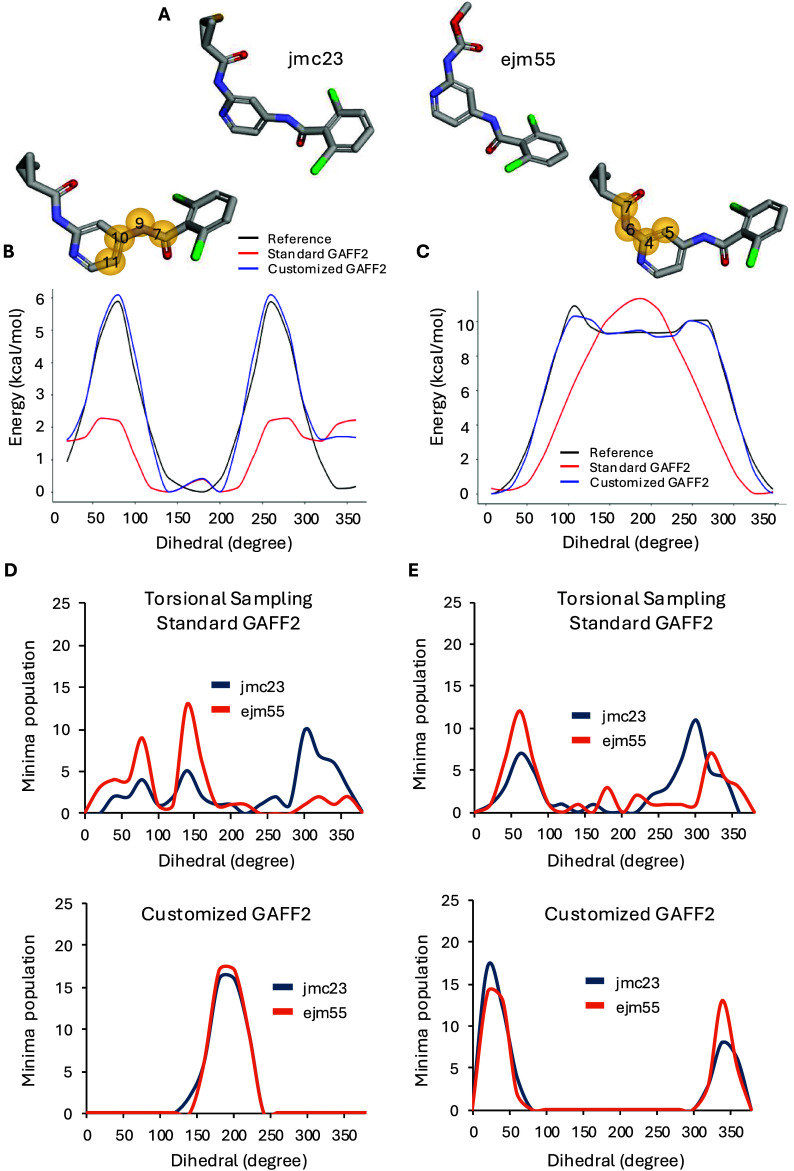
Comparative torsional sampling analysis between standard GAFF2 and customized GAFF2 for the jmc23-ejm55 ligand transformation (TYK2 system) in aqueous solution, an example where customized GAFF2 improves RBFE accuracy. (A) Chemical structures of ligands jmc23 and ejm55 show structural variations. (B–C) Reference torsional energy profiles (black) and corresponding profiles generated with standard GAFF2 (red) and customized GAFF2 (blue) for torsions 7–9–10–11 (B) and 7–6–4–5 (C). Insets show the reparameterized ligand structure indicating the torsions analyzed. (D, E) Torsional sampling distributions of torsions 7–9–10–11 (D) and 7–6–4–5 (E) comparing standard GAFF2 (top panel) and customized GAFF2 (bottom panel) for ligands jmc23 and ejm55, illustrating the population distributions across different PES minima. All panels report the dihedral angle scale in degrees (*X*-axis) using the absolute torsion values, with the minimum located at its corresponding dihedral angle.

Overall, this analysis demonstrates that torsion reparameterization improves RBFE estimates by significantly redefining the ligand potential energy surface in agreement with high-quality quantum mechanical reference data, thereby modulating conformational accessibility in both end-states and enhancing sampling quality. For the investigated cases, the reparameterization also leads to increased overlap between ligand conformational distributions, which facilitates sampling, leading to smoother alchemical free-energy pathways, thus reducing error bars of RBFE predictions. However, these conclusions are drawn exclusively from the present data set involving the TYK2 and MCL1 systems. Further validation across additional ligand transformations and protein systems will be required to draw more general conclusions. In particular, while good torsional parameters are essential, there are other factors that influence the quality of RBFE estimates, including nonbonded interactions, charge models, and error cancellation. Importantly, AFFDO’s value extends beyond RBFE to other free energy estimations, such as absolute binding free energies (ABFE) and solvation free energies, which could directly benefit from enhanced PES descriptions of individual ligands.

### 3.3 Timing and Computational Cost of Torsion Optimization with AFFDO

In Table 2, we report wallclock times taken for each step of the AFFDO workflow using a fragment (out of the two available) from jmc28 and L35 ligand parametrization. The most time-consuming steps include centroid optimization (due to QC computations), torsional scans (again due to QC), and parameter fitting (due to a series of torsional scans needed during gradient-based optimization).

**Table 2:** Wall Time (Seconds) Taken from AFFDO Workflow Steps during Torsion Reparameterization of jmc28_F1 and L35_F1 Ligand Fragments (See Figure 2) on a Cloud Instance with 36 AMD EPYC 7282 CPU cores, 4 NVIDIA RTX4090 GPUs, and 64 GB Memory

	Wall time (s)	
Step	jmc28_F1 (1 torsion)	L35_F1 (3 torsions)
1. Ligand fragmentation	<1	<1
2. Conformer generation	4	4
3. Conformer minimization (XTB)	27	148
4. Clustering	<1	<1
5. Centroid optimization (QC and MM)	900	2600
6. Significant minima selection	<1	<1
7. Significant torsion selection	<1	<1
8. Torsional scan (XTB and QC)	1742	22995
9. Fitting torsion parameters	133	623
10. Parmed input file generation	<1	<1
Total run time	2807	26370

Although the computational cost of torsion optimization using AFFDO can be high (47 min for jmc28_F1 and 7.3 h for L35_F1), RBFE computations themselves are significantly more time-consuming (approximately 20–24 h per MCL1 transformation and over 48 h per TYK2 transformation on identical GPU hardware); these runtimes correspond to the full triplicate campaign, i.e., three independent RBFE trials per transformation. The additional cost for torsion optimization is thus easily justifiable and further is inherently parallelizable across scanned points. Estimating the total time for reparameterizing a molecule based purely on its size or number of torsions is challenging since QC torsional scan durations depend significantly on the complexity of the conformational energy landscape, convergence of the constrained geometry optimization and SCF procedures, which can vary considerably.

Furthermore, the associated wallclock times can be reduced in a number of ways. In general, providing more CPU cores for xtb calculations and employing a multilevel parallelism can be helpful. Similarly, providing more GPUs for QC calculations would reduce the wall time spent on torsional scans. For xtb runs reported in this study, we used all available CPU cores in the cloud instance but torsional scan calculations were launched one after another. Instead, launching all scans simultaneously with multiple CPU cores for each calculation could lead to better performance overall. QC calculations were launched in batches of 4, utilizing all 4 GPUs for 4 different calculations. Availability of more GPUs will allow increasing this batch size and therefore speed up the procedure. Additionally, in this study, we used conservative cutoffs for QC calculations (10^–7^ *a.u*. density matrix cutoff for self-consistent field (SCF) convergence, 10^–12^ *a.u*. integral cutoff, 10^–8^ exchange correlation cutoff, and default geometry optimization cutoffs) and 20° intervals for torsional scans. Loosening these cutoffs and carrying out scans with more relaxed angular intervals (e.g., 30°), which is configurable in AFFDO, can lead to significant performance gains.

## 4 Conclusions and Future Directions

We have developed a new computational infrastructure called AFFDO that facilitates force field development and optimization. In particular, the current version enables the reparameterization of GAFF/GAFF2 torsion parameters for drug-like molecules. Benchmarks demonstrated that AFFDO improves agreement with QC torsion-scan reference data (by ∼0.8 kcal/mol RMSE across the benchmark torsion-scan data set^27^) in an automated manner and within a reasonable computational time using cloud computing resources. RBFE simulations using standard GAFF2 versus reparameterized GAFF2 showed that AFFDO-optimized torsions reduced overall prediction errors for MCL1 (by ∼0.5 kcal/mol RMSE), whereas the aggregate improvement for TYK2 was smaller (by ∼0.2 kcal/mol RMSE) and not statistically significant at the full-network level, indicating that for the TYK2 benchmark, RBFE accuracy may be limited by model deficiencies beyond torsion descriptions.

AFFDO can be further improved in terms of usability and performance. The overall runtime of the reparameterization process is currently dominated by reference data generation; this cost could be reduced by enabling support for multiple cloud instances, which is not possible at the moment. Runtime could also be substantially reduced by leveraging fast AI-based models for torsional-scan reference data generation, such as the QDπ2 model,^67–69^ along with storing reference data in a database^70^ so that it can be reused when reparameterizing new ligands. Finally, we will endeavor to expand the scope of our force field parameter optimization infrastructure to include improved nonbonded (e.g., Lennard-Jones), electrostatic, and linear-response models.

## Supplementary Material





## Data Availability

AFFDO is available as a web service at https://www.attmosdiscovery.com/tools free of charge, where users can submit jobs and access results via a job ID (including an in-browser report with torsion plots) and downloadable outputs. Input files used for the benchmarks in this work (both AFFDO runs and the RBFE workflow) are provided in the Supporting Information.

## Notes

Torsion plots, performance evaluation of AFFDO torsional parametrization across additional systems, technical details of RBFE simulations, RBFE predictions, and sampling investigation (PDF)
Input files for AFFDO torsion reparameterization runs and the RBFE simulations reported in this work (ZIP)
