# Supplementary material for: Automated Force Field Developer and Optimizer Platform: Torsion Reparameterization
Source: J Chem Inf Model. 2026 Mar 9;66(6):3206–19. doi: 10.1021/acs.jcim.6c00528 (PMC13014461; doi:10.1021/acs.jcim.6c00528)
Supplement: Supplementary file 1 [file ci6c00528_si_001.pdf]

Supporting Information
Automated Force Field Developer and Optimizer Platform: Torsion Reparameterization

Alejandro Blanco-Gonzalez,[†] William Betancourt,[‡] Ryan Michael Snyder,[¶] Shi Zhang,[¶] Timothy J. Giese,[¶] Zeke A. Piskulich,[¶] Andreas W. Götz,[*,§,†] Kenneth M. Merz, Jr.,[*,‖,†] Darrin M. York,[*,¶,†] Hasan Metin Aktulga,[*,‡,†] and Madushanka Manathunga[*,†]

†*ATTMOS Inc., 325 E Grand River Ave Ste 328, East Lansing, Michigan 48823, United States*

‡*Department of Computer Science and Engineering, Michigan State University, East Lansing, Michigan 48824-1322, United States*

¶*Laboratory for Biomolecular Simulation Research, Center for Integrative Proteomics Research and Department of Chemistry and Chemical Biology, Rutgers University, Piscataway, New Jersey 08854-8087, United States*

§*San Diego Supercomputer Center, University of California San Diego, La Jolla, California 92093-0505, United States*

‖*Department of Chemistry and Department of Biochemistry and Molecular Biology, Michigan State University, East Lansing, Michigan 48824-1322, United States*

E-mail: agoetz@sdsc.edu; merz@chemistry.msu.edu; Darrin.York@rutgers.edu; hma@attmosdiscovery.com; madu@attmosdiscovery.com

Contents

- S1: Torsion plots
- S2: Performance Evaluation of AFFDO Torsional Parameterization Across Additional Systems
- S3: Technical details of RBFE simulations
- S4: RBFE predictions
- S5: Sampling investigation

S1: Torsion plots

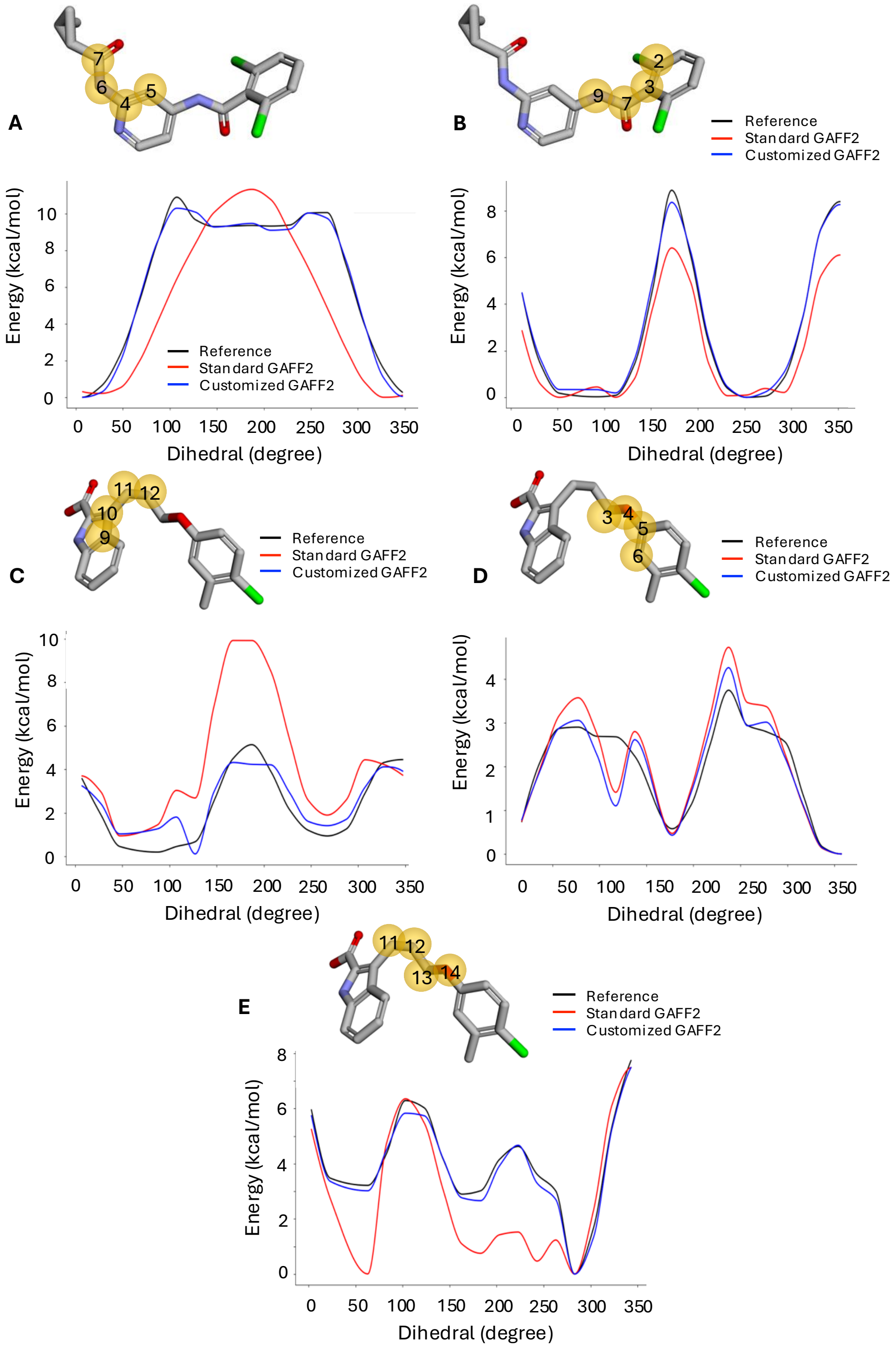

Figure S1: Additional torsional scan energy profiles for jmc28 ligand from TYK2 (**A**, **B**) and L35 ligand from MCL1 protein system (**C**, **D**, **E**). The corresponding torsions are highlighted in the molecular structures by orange spheres. The customized GAFF2 profiles (in blue) show closer alignment with the reference data than the standard GAFF2 profiles (in red), highlighting AFFDO's enhanced accuracy in describing torsions.

We note that an independent TYK2 torsion-scan dataset has recently been reported using ωB97M-D3(BJ)/def2-TZVPPD reference scans within an OpenFF/Torpenter refitting workflow.[1] After accounting for dihedral-angle conventions, a qualitative visual cross-check for the overlapping ligand TYK2-jmc28 (main-text Fig. 3A–B and SI Fig. S1A–B) shows consistent locations of the dominant minima and major barrier regions, as expected, despite differences in the force-field framework and QM reference level. We also include the AFFDO run outputs for the analyzed TYK2 and MCL1 fragments as part of the supporting material submission package.

S2: Performance Evaluation of AFFDO Torsional Parameterization Across Additional Systems

To further assess the accuracy of AFFDO's torsional parameterization, we extended the analysis beyond the TYK2 and MCL1 ligand systems by evaluating additional ligand systems such as CDK2, JNK1, P38α, and Thrombin from the publicly available dataset.[2] This extended evaluation provides a broader validation of AFFDO's ability to refine force field torsional parameters across diverse chemical environments.

We systematically compared AFFDO customized GAFF2 and standard GAFF2 against reference torsional scans computed at the DFT level (PBE0-D3BJ/6-31G* for neutral and cationic molecules, PBE0-D3BJ/6-31+G* for anionic molecules), ensuring a direct assessment independent of relative binding free energy (RBFE) calculations. The dataset includes 126 unique ligands, comprising 269 molecular fragments and spanning 466 individual torsions. This analysis quantitatively evaluates the agreement between force field-derived and quantum chemistry (QC) torsional energy profiles using root-mean-square error (RMSE), mean absolute error (MAE), and correlation metrics.

For each torsion, RMSE and MAE were computed over 18 scan points, where at each point the target torsion was constrained while the remaining degrees of freedom were relaxed via

constrained geometry optimizations. The relative energies from both MM methods (standard GAFF2 and AFFDO customized GAFF2) were directly compared against the QC (DFT) reference, with uncertainties expressed as 95% confidence intervals calculated analytically from the standard error of the mean across all torsions and systems. Similarly, Pearson and Spearman correlation coefficients were calculated to assess the overall agreement in the energy profiles, and RMSD values between MM-optimized and QC-optimized geometries were computed to evaluate structural agreement.

Table S1 shows how AFFDO consistently improves the reproduction of reference torsion scans across all evaluated systems. For clarity, these statistics quantify agreement of QC torsion scan energy profiles evaluated over dihedral-scan grid points, and should not be interpreted as RBFE predictive accuracy. Compared to standard GAFF2, AFFDO reduces RMSE from 1.48 kcal/mol to 0.65 kcal/mol, and MAE from 1.12 kcal/mol to 0.49 kcal/mol, demonstrating a substantial enhancement in torsional accuracy. Furthermore, Pearson and Spearman correlation coefficients increase from 0.80 to 0.91 and from 0.79 to 0.90, respectively, indicating a significantly improved alignment with QC energy landscapes. Additionally, geometry optimizations performed with AFFDO-derived parameters yield comparable or better root-mean-square deviations (RMSD) relative to QC-optimized structures, reinforcing the reliability of the parameterization.

The improvements in accuracy are particularly pronounced in ligands with complex torsional landscapes, where standard GAFF2 struggles to correctly capture energy barriers and minima locations. To illustrate these advancements, Figure S2 presents selected torsional scan energy profiles that compare the QC reference data, standard GAFF2 and AFFDO-optimized parameters between representative ligands of each system. These results confirm that AFFDO consistently improves PES accuracy over standard GAFF2 in torsion parameterization across diverse systems, extending its effectiveness beyond TYK2 and MCL1.

Table S1: GAFF2 vs AFFDO performance parameters evaluation across several systems (126 ligands) of a public dataset.[2] Metrics quantify agreement with QC torsional potential energy surfaces obtained from constrained dihedral scans evaluated on a 20° angular grid (i.e., scan-point energies) and should not be interpreted as RBFE predictive accuracy.

**Metric**	**GAFF2**	**AFFDO**
MAE[a] (kcal/mol)	1.12 ± 0.07	0.49 ± 0.04
RMSE[a] (kcal/mol)	1.48 ± 0.10	0.65 ± 0.05
Pearson (r) (unitless)	0.80	0.91
Spearman (ρ) (unitless)	0.79	0.90
Max RMSD[b] (Å)	0.82	0.80

[a] Uncertainties are reported as 95% CI calculated analytically.
[b] RMSD values measure conformational deviations relative to QC-optimized structures.

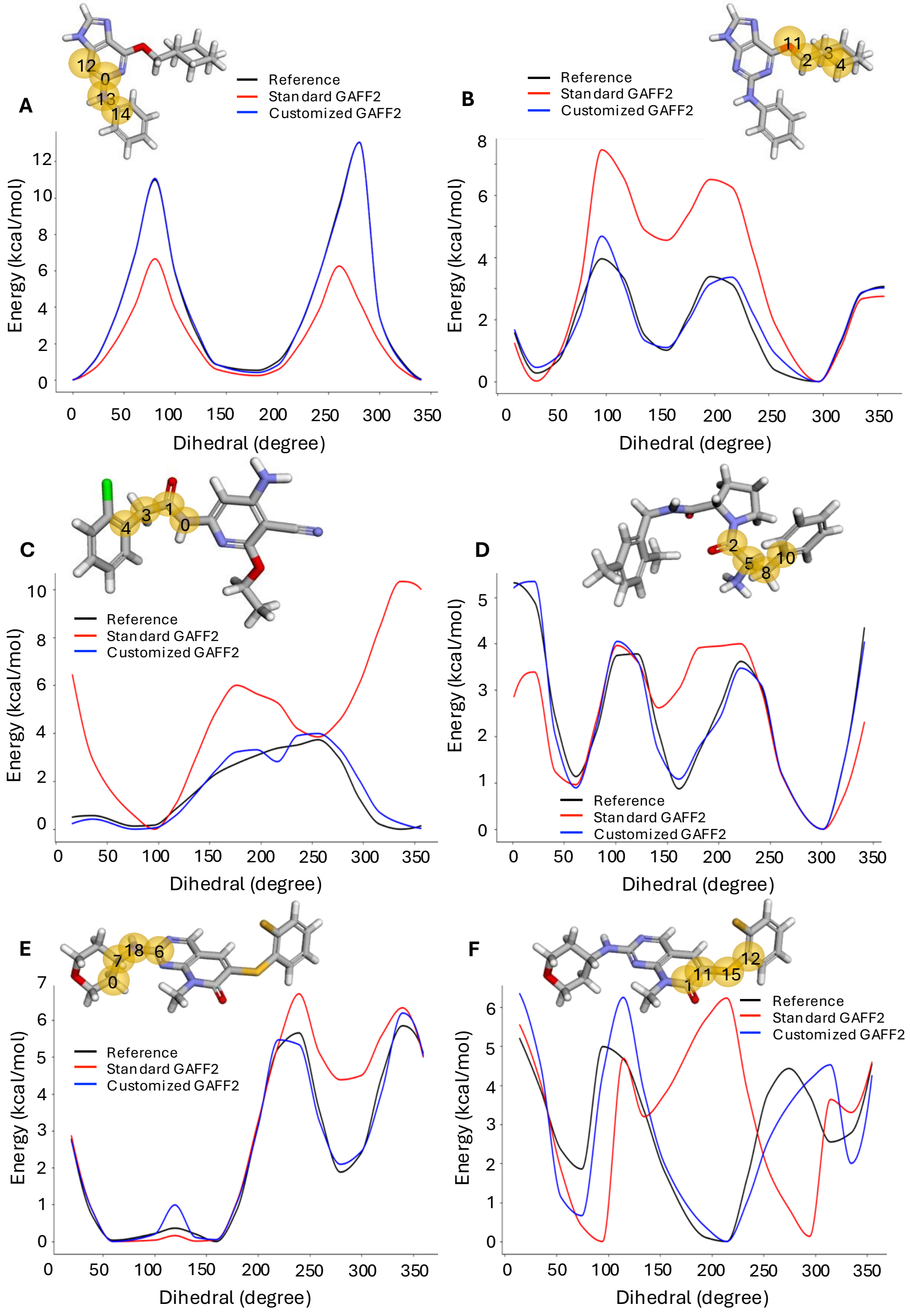

Figure S2: Torsional scan energy profiles for representative ligands from the database[2] illustrating AFFDO's enhanced accuracy. Panels (**A**) and (**B**) show two torsional scans for the *1h1q* ligand (CDK2); (**C**) the scan for the *18625-1* ligand (JNK1); (**D**) the scan for the *7a* ligand (Thrombin); and (**E**) and (**F**) scans for two torsions of the *2h* ligand (P38α). Torsions are highlighted by orange spheres. Customized GAFF2 profiles (blue) align more closely with QC reference data (black) than standard GAFF2 (red), underscoring improved accuracy and transferability.

S3: Technical details of RBFE simulations

To setup RBFE simulations, the protein and ligand structures were obtained from publicly available dataset and used as input to the ProFESSA workflow.[3] Here AMBER ff19SB force field,[4] GAFF2[5] and TIP4P-Ew[6] were used for proteins, ligands and water molecules. All RBFE simulations of a given protein were prepared from a single ProFESSA run to ensure that all solvated MD boxes have the same number of water molecules and ions. The RBFE transformations were performed with 21 λ windows (0.00, 0.05, 0.10, 0.15, 0.20, 0.25, 0.30, 0.35, 0.40, 0.45, 0.50, 0.55, 0.60, 0.65, 0.70, 0.75, 0.80, 0.85, 0.90, 0.95, 1.0) using the SSC(2) smoothstep softcore function.[7] All simulations were performed with a 1 fs time step, and electrostatics were treated with the particle mesh Ewald method[8,9] using a 10 Å non-bonded interaction cutoff, a 1 pt/Å reciprocal space grid, and tinfoil boundary conditions. The temperature was regulated with the Langevin thermostat using a friction constant of 2.0 ps^{-1}. The pressure was regulated with a Monte Carlo barostat.

The ProFESSA workflow prepared the systems using a 2-state simulation setup that minimizes and equilibrates the 2 end-states in a series of steps involving heating, pressure relaxation, simulated annealing, and gradual release of harmonic restraints on the protein. This is followed by equilibration of each intermediate alchemical state departing from the nearest end-state for 2.4 ns in the NPT ensemble at 300 K and 1 atm. Production sampling was performed for 5 ns with Hamiltonian replica exchange in the NPT ensemble at 300 K and 1 atm. Exchange attempts were made every 20 ps. The production simulations were repeated 3 times with different thermostat random seed numbers. A summary of the end-state equilibration procedure is described here, and further information can be found in Ref. 3. In the complex phase, "real" state structures, i.e. $\lambda = 0, 1$ underwent energy minimization with the ligands and receptor restrained by a 5 kcal/mol/Å RMSD restraint. Energy minimization is then performed without the restraint. The system is heated from 5 to 300 K over 5 ps with an 5 kcal/mol/Å RMSD restraint on the solute and receptor. This is repeated with a new set of initial velocities. The end-states are then allowed to equilibrate

for 500 ps at constant pressure. End-states are then run for 500 ps towards a target volume, achieved through semi-isotropic pressure scaling on the system's solvent box. Each end-state is again heated from 5 to 300 K over 500 ps with a 5 kcal/mol/Å RMSD restraint on the solute and receptor, then allowed to equilibrate with the restraints for 500 ps under NPT conditions. End-states are then heated from 300 to 600 K over 50 ps, equilibrated at 600 K over 100 ps, then cooled to 300 K over 50 ps. The system is then equilibrated for 200 ps at 300 K under NPT conditions with a 2, 1, 0.5, 0.25, 0.1 kcal/mol restraint, then further equilibrated for 200 ps without any restraint.

Common-core regions for each RBFE edge were determined using the single fragment maximum common substructure, extended to exclude atoms undergoing changes in hybridization or atomic number. The common core is then reduced to ensure each ligand in each edge shares the same common-core region across the network. Electrostatics, 1-4 nonbonding terms and torsion terms are scaled in both the softcore region and at the interface between the common-core and soft-core regions. van der Waals interactions between the soft-core and common core regions are scaled but maintained within the softcore region. Soft-core regions are modeled using a second-order smooth-step soft-core potential.[7]

Error evaluation. As stated above, production simulations were repeated 3 times to produce independent estimates of the transformation free energies in the protein-ligand complex $\Delta G_{\mathrm{com},i} \pm \delta\Delta G_{\mathrm{com},i}$ and aqueous environments $\Delta G_{\mathrm{aq},i} \pm \delta\Delta G_{\mathrm{aq},i}$ for each trial, i. The uncertainty in the free energy of each alchemical state is calculated from cyclic moving block bootstrap analysis, and these errors are propagated to estimate the transformation free energy uncertainties. The error estimates made from a single trial are an underestimate of the true error due to the finite length of the simulation. Another method for estimating errors is the "ensemble average approach", which calculates a series of results from independent trials that differ by their initial conditions (such as thermostat random seed), and the uncertainty in the result is the standard error of the mean. In the present case, the RBFE production simulations were repeated 3 times to obtain 3 estimates in each environment: $\Delta G_{\mathrm{com},1} \pm$

$\delta\Delta G_{\text{com},1}$, $\Delta G_{\text{com},2} \pm \delta\Delta G_{\text{com},2}$, $\Delta G_{\text{com},3} \pm \delta\Delta G_{\text{com},3}$ and $\Delta G_{\text{aq},1} \pm \delta\Delta G_{\text{aq},1}$, $\Delta G_{\text{aq},2} \pm \delta\Delta G_{\text{aq},2}$, $\Delta G_{\text{aq},3} \pm \delta\Delta G_{\text{aq},3}$. The RBFE is the difference in the means

$$\Delta\Delta G = \langle \Delta G_{\text{com}} \rangle - \langle \Delta G_{\text{aq}} \rangle, \tag{1}$$

and the error is given by

$$\delta\Delta\Delta G = \sqrt{\delta\langle \Delta G_{\text{com}} \rangle^2 + \delta\langle \Delta G_{\text{aq}} \rangle^2}, \tag{2}$$

where

$$\delta\langle \Delta G_{\text{env}} \rangle^2 = \frac{1}{3} \frac{\sum_{i=1}^{3} (\Delta G_{\text{env},i} - \langle \Delta G_{\text{env}} \rangle)^2}{3-1} + \frac{1}{3} \sum_{i=1}^{3} \delta\Delta G_{\text{env},i}^2. \tag{3}$$

The first term is the (squared) standard error of the independent estimates, and the second term is the average (squared) bootstrap error. Our practical experience is that the errors are dominated by the variation between independent trials rather than the observed fluctuations within a single trial.

S4: RBFE predictions

To further assess the impact of AFFDO-optimized torsion parameters beyond direct comparisons with quantum chemical (QC) reference data, we evaluated their influence on Relative Binding Free Energy (RBFE) simulations. This section systematically examines the key factors influencing the results, statistical trends observed, and broader methodological considerations.

While RBFE calculations (Table S2) provide an indirect means of evaluating torsion parameterization improvements, they introduce additional complexities such as conformational sampling, end-state overlap, and nonbonded interactions that may confound the direct effects of torsional refinement. Therefore, a dedicated analysis assessing the agreement between AFFDO-optimized parameters and QM reference data was performed independently

(see Section S2). This direct evaluation isolates the improvements in ligand potential energy surfaces (PES) from the complexities of RBFE calculations, offering a clearer assessment of AFFDO's impact.

The ligand transformations included in this study were selected based on cases where standard GAFF2 shows greater deviations from experimental values. In fact, some of the transformations within the TYK2 system, in particular many that involve the ligands ejm55 or ejm31 (e.g., jmc23-ejm55, ejm47-ejm55, ejm55-ejm54, or ejm31-ejm46), have been previously reported to be particularly challenging or outliers in the RBFE calculations.[3,10] These cases represent scenarios where *a priori* parameter refinement could have the most pronounced impact. By focusing on these transformations, we aimed to assess whether improvements in torsion parameterization could alleviate some of the limitations associated with standard GAFF2 in these systems.

Table S2: Experimental and computed RBFE values using standard GAFF2 and AFFDO-optimized GAFF2 for TYK2 and MCL1 ligand transformations. Experimental values from Refs.[2,11]

**$\Delta\Delta G_b$ for TYK2**			
Ligand transformation	Experimental (kcal/mol)	GAFF2 (kcal/mol)[a]	AFFDO (kcal/mol)[a]
ejm42-ejm55	0.57	-0.93 ± 0.08	-0.91 ± 0.04
jmc28-jmc30	0.07	-0.71 ± 0.03	-0.02 ± 0.08
jmc28-jmc27	-0.30	-0.79 ± 0.02	-0.48 ± 0.02
jmc23-jmc30	0.76	1.82 ± 0.07	2.25 ± 0.06
jmc23-ejm55	2.49	-0.04 ± 0.12	0.79 ± 0.08
ejm42-ejm54	-0.75	-2.48 ± 0.07	-2.23 ± 0.07
ejm44-ejm55	-1.79	-3.07 ± 0.13	-2.93 ± 0.06
ejm49-ejm31	-1.79	-0.03 ± 0.12	−0.52 ± 0.08
ejm49-ejm50	-1.23	-0.45 ± 0.10	-0.44 ± 0.10
ejm55-ejm54	-1.32	-0.75 ± 0.06	-1.22 ± 0.04
ejm31-ejm46	-1.77	-0.81 ± 0.07	-1.03 ± 0.02
ejm31-jmc28	-1.44	-0.77 ± 0.19	0.62 ± 0.12
ejm31-ejm48	0.54	0.23 ± 0.06	0.16 ± 0.06
ejm42-ejm48	0.78	-0.61 ± 0.11	-0.37 ± 0.09
ejm47-ejm55	0.49	−1.35 ± 0.06	−0.17 ± 0.07
MAE[b]		1.18 ± 0.29	0.98 ± 0.31
RMSE[b]		1.32 ± 0.30	1.15 ± 0.28
Max. Error		2.53	2.06
R^2 (unitless)		0.17	0.30
Spearman (ρ) (unitless)		0.36	0.61
Kendall (τ) (unitless)		0.29	0.44
Wilcoxon signed-rank p-value[c]		0.073	
Paired bootstrap ΔRMSE (95% CI)[d]		0.18 (95% CI: −0.16, 0.44)	
**$\Delta\Delta G_b$ for MCL1**			
L27-L33	-0.76	-1.54 ± 0.13	-1.51 ± 0.19
L35-L67	1.23	-2.15 ± 0.47	-1.89 ± 0.08
L35-L53	-1.15	-3.66 ± 0.29	-3.01 ± 0.10
L35-L60	-0.10	-2.26 ± 0.22	-1.91 ± 0.04
L38-L35	-1.79	-1.66 ± 0.19	−1.84 ± 0.06
L49-L67	0.78	-1.99 ± 0.17	0.47 ± 0.23
L65-L67	0.83	1.21 ± 0.02	1.09 ± 0.07
L67-L27	1.46	3.65 ± 0.42	3.70 ± 0.07
L67-L32	1.00	4.66 ± 0.34	2.76 ± 0.19
L67-L37	-1.37	1.30 ± 0.06	1.15 ± 0.05
L67-L50	-1.75	1.56 ± 0.14	0.34 ± 0.04
L67-L52	-1.64	0.74 ± 0.11	0.57 ± 0.09
L67-L58	-1.83	-1.41 ± 0.04	−1.47 ± 0.08
L67-L63	-1.48	-1.45 ± 0.24	−1.44 ± 0.12
MAE[b]		1.91 ± 0.64	1.39 ± 0.53
RMSE[b]		2.28 ± 0.50	1.71 ± 0.41
Max. Error		3.66	3.12
R^2 (unitless)		0.10	0.23
Spearman (ρ) (unitless)		0.07	0.28
Kendall (τ) (unitless)		0.03	0.25
Wilcoxon signed-rank p-value[c]		0.00085	
Paired bootstrap ΔRMSE (95% CI)[d]		0.56 (95% CI: 0.21, 0.98)	

[a] uncertainties reported as per-transformation standard errors from simulation (3-trials).
[b] uncertainties reported as the bootstrapped 95% CI (1000 samples).
[c] paired Wilcoxon signed-rank test on ΔMAE (GAFF2−AFFDO) from per-transformation absolute errors; improvement if $p < 0.05$.
[d] paired bootstrap on ΔRMSE (GAFF2−AFFDO); 95% CI (1000 resamples); improvement if CI excludes 0.

Given the context of our selection, Table S2 reveals notable improvements in individual cases after torsional reparameterization with AFFDO. Among others, the TYK2 transformations previously mentioned. Across both the TYK2 and MCL1 systems, approximately 80% of the transformations showed improved RBFE estimates compared to standard GAFF2. Within this subset, the average reduction error (MAE/RMSE) was approximately ∼0.4 kcal/mol for TYK2 and ∼0.6 kcal/mol for MCL1 (see Table S3 for a breakdown of these cases), with the largest observed improvement reaching 2.45 kcal/mol for transformation L49-L67. These findings reinforce the ability of AFFDO to refine torsional profiles and improve sampling consistency, ultimately benefiting alchemical free-energy calculations.

However, not all transformations exhibited improvement, and the resulting RBFE estimates remained within the error margins of standard GAFF2. In some cases, such as L67–L27 (MCL1), persistent sampling discrepancies contributed to limited gains. In others, like ejm49–ejm50 (TYK2), the customized torsional parameters did not significantly alter the conformational space already sampled by standard GAFF2, thereby limiting the impact of reparameterization. These factors help explain the negligible or modest improvements observed in certain transformations (see detailed discussion in Section S5). One outlier transformation where AFFDO worsens the RBFE prediction with respect to experiment compared to the standard GAFF2 is ejm31-jmc28. This transformation had the largest estimated standard error of the AFFDO results for this target, and likely are due to sampling issues.

To further examine the influence of parameterization in these cases, we repeated a subset of the RBFE simulations using torsional parameters optimized on alternative ligands, including those directly involved in the transformations. These additional tests focused on transformations where the original parameters, derived from jmc28 (TYK2) or L35 (MCL1), had led to negligible improvements over GAFF2. Interestingly, while most of the new simulations reproduced the previously observed outcomes (e.g., L67–L27, or ejm49–ejm50 showed no significant changes), certain cases, such as ejm49–ejm31 and L65–L67, exhibited modest

improvements in RBFE accuracy with the alternative parameter set. These findings suggest that, although parameterizing a single representative ligand is generally a cost-effective and practical strategy within a congeneric series, exploring additional parametrizations in select cases may help improve outcomes for specific transformations where initial improvements were limited.

This variability highlights the complexity of relative binding free energy (RBFE) methods, where improved torsional descriptions alone do not always translate directly into better agreement with experimental values, as additional factors, including charge models, non-bonded interactions, and end-state sampling, contribute to overall prediction accuracy. Therefore, when considering the entire set of transformations, including cases with negligible improvements, the overall improvements in MAE and RMSE across all transformations decrease. We observe an overall error reduction (MAE/RMSE) of ∼0.5 kcal/mol for MCL1, and a modest reduction of ∼0.2 kcal/mol for the TYK2 system that is not statistically significant under the paired tests reported in Table S2 (Wilcoxon $p > 0.05$ and paired bootstrap CI including 0). The TYK2 outcome reflects the high complexity of that system and the fact that several of its ligand transformations remain particularly challenging for RBFE methodologies, even after torsional parameter optimization.

While these aggregate statistics may appear limited, they should be interpreted within the broader context of the study's focus on challenging transformations, where standard GAFF2 exhibits known deviations from experiments. Consequently, while AFFDO enhances torsional accuracy and improves free-energy pathway smoothness in most cases, its impact is inherently limited by model deficiencies beyond torsion descriptions. Beyond RBFE applications, AFFDO's improvements in torsional profiles could also potentially impact other free-energy methods such as Absolute Binding Free Energy (ABFE) and solvation-free energy calculations, where an accurate representation of ligand potential energy surfaces is equally critical.

Table S3: Statistical metrics that compare standard GAFF2 and AFFDO customized GAFF2 parameters in transformations benefiting from AFFDO torsional reparametrization for TYK2 and MCL1.

	TYK2	
**Metric**	**GAFF2 (kcal/mol)**	**AFFDO (kcal/mol)**
MAE[a]	1.35 ± 0.36	0.91 ± 0.33
RMSE[a]	1.47 ± 0.35	1.07 ± 0.28
Max Error	2.53	1.70
Wilcoxon signed-rank p-value[b]	0.00098	
Paired bootstrap ΔRMSE (95% CI)[c]	0.40 (95% CI: 0.20, 0.61)	
	**MCL1**	
MAE[a]	2.04 ± 0.68	1.43 ± 0.56
RMSE[a]	2.38 ± 0.57	1.73 ± 0.50
Max Error	3.66	3.12
Wilcoxon signed-rank p-value[b]	0.00049	
Paired bootstrap ΔRMSE (95% CI)[c]	0.65 (95% CI: 0.28, 1.10)	

[a] uncertainties reported as the bootstrapped 95% CI (1000 samples).
[b] paired Wilcoxon signed-rank test on ΔMAE (GAFF2−AFFDO) from per-transformation absolute errors; improvement if $p < 0.05$.
[c] paired bootstrap on ΔRMSE (GAFF2−AFFDO); 95% CI (1000 resamples); improvement if CI excludes 0.

Correlation Metrics, Precision and Reported Uncertainties. While RMSE and MAE provide a direct measure of error reduction, Pearson's correlation coefficient (r) and Spearman's rank correlation coefficient (ρ), included as complementary metrics, should be interpreted with caution, especially when transformations are not systematically mapped within a well-connected perturbation network. Outliers and transformation-specific challenges can strongly influence correlation values. Therefore, RMSE and MAE remain the primary metrics for assessing predictive accuracy in RBFE contexts.[12] It should also be noted that the reported statistical uncertainties incorporate variability across three independent RBFE trials performed using the ProFESSA workflow. These uncertainties are derived from the combined variance across independent repeats and bootstrap-derived fluctuations within individual simulations. Notably, the observed reduction in statistical uncertainty with AFFDO parameters is attributed to the introduction of well-defined torsional barriers, which confine sampling to specific minima, enhancing reproducibility across independent trials. However, it is important to distinguish statistical reproducibility from absolute methodological accuracy, as systematic errors unrelated to torsion parameterization may

persist.

To assess whether differences between GAFF2 and AFFDO are statistically significant, we use paired tests because both methods are evaluated on the *same* set of ligand transformations. We therefore report (i) a paired Wilcoxon signed-rank test on per-transformation absolute errors (i.e., ΔMAE (GAFF2 − AFFDO)) and (ii) a paired bootstrap analysis on ΔRMSE (GAFF2 − AFFDO), where transformations are resampled with replacement and RMSE is recomputed for both methods on the same resampled set to obtain a distribution of paired differences. We report the 95% confidence interval from 1000 resamples; improvement is indicated when the CI excludes 0. A paired Student's t-test yields consistent conclusions and is therefore not shown.

S5: Sampling investigation

Table S2 presents a detailed comparison of computed relative binding free energy (RBFE) values using standard GAFF2 and customized GAFF2 force fields across various ligand transformations within the TYK2 and MCL1 protein systems. The torsion parameter optimization was initially carried out using representative ligands jmc28 (TYK2) and L35 (MCL1), and the resulting parameters were subsequently transferred to the other ligands involved in the alchemical transformations examined in this study. Interestingly, while customized GAFF2 improved RBFE predictions in several cases, some transformations exhibited negligible or no improvement over the standard GAFF2 predictions.

To gain deeper insights into these outcomes, we investigated the underlying torsional sampling patterns during free-energy simulations, specifically focusing on transformations showing the most pronounced differences. Figures 5 (in the main text), S3, and S4 illustrate these torsional sampling patterns for transformations in aqueous solution, as significant differences were not observed within the protein environment. This analysis intentionally targeted transformations where the impact of the parameterization on the RBFE was most

significant (jmc23-ejm55 for TYK2 and L49-L67 for MCL1) and transformations where the impact was minimal or absent (ejm49-ejm50 for TYK2 and L67-L27 for MCL1).

A clear example illustrating the impact of torsion reparameterization is shown in Figure 5B (torsion 7-9-10-11). Here, AFFDO substantially increased the energy barrier height compared to GAFF2, clearly defining distinct minima and significantly narrowing the accessible conformational space. As a direct consequence, the sampling distributions for both ligands involved in the transformation became more focused and overlapped substantially in the AFFDO customized GAFF2 scenario (Figure 5D). Such an enhanced overlap translates into smoother alchemical transitions and, in this case, also notably improved RBFE estimates. Another illustrative case is shown in Figure 5C (torsion 7-6-4-5), where reparameterization shifted the torsion phase and slightly modified barrier heights, resulting in a more defined and unified minima region. This modification leads to a more consistent conformational sampling across both end-states, thereby alleviating the complexity inherent to alchemical free-energy calculations and ultimately enhancing the reliability of RBFE predictions.

Improved torsion fittings enable a more accurate description of relative internal conformational energies and their representative populations in a simulation. Torsion fitting will only be expected to improve results if it results in a significant difference in the observed populations of distinct conformational states in the simulation. For example, in the ejm49–ejm50 transformation (Figure S3), torsion 2–3–7–9 retained an almost invariant profile between standard and customized GAFF2. As a result, the overall conformational landscape remained largely unaffected and consequently the RBFE estimates from the customized force field were consistent with those from standard GAFF2 (see Table S2). Likewise, for torsion 7–6–4–5, a slight shift in phase and minima was observed, but this effect was not sufficient to significantly impact the distribution of sampled conformational states. Consistent observations were made for the MCL1 system (Figure S4). Reparameterization of torsion 11–12–13–14 refined the torsional profile by increasing the barrier height and slightly shifting the minima, which in the L49–L67 transformation enhanced sampling of relevant conforma-

tions and improved the RBFE estimates. In contrast, for L67–L27, the reshaped potential energy surface did not substantially alter the conformational populations, resulting in RBFE values comparable to those obtained with the standard GAFF2 parameters.

However, custom torsion angle fitting alone still does not guarantee improved RBFE estimates given there are other important terms in the force field that are coupled and collectively contribute to binding. Nonetheless, creating force field models for flexible drug-like molecules that have accurate relative conformational energies (and barriers) through torsion fitting is a critical step toward improving the predictive capability of the calculations.

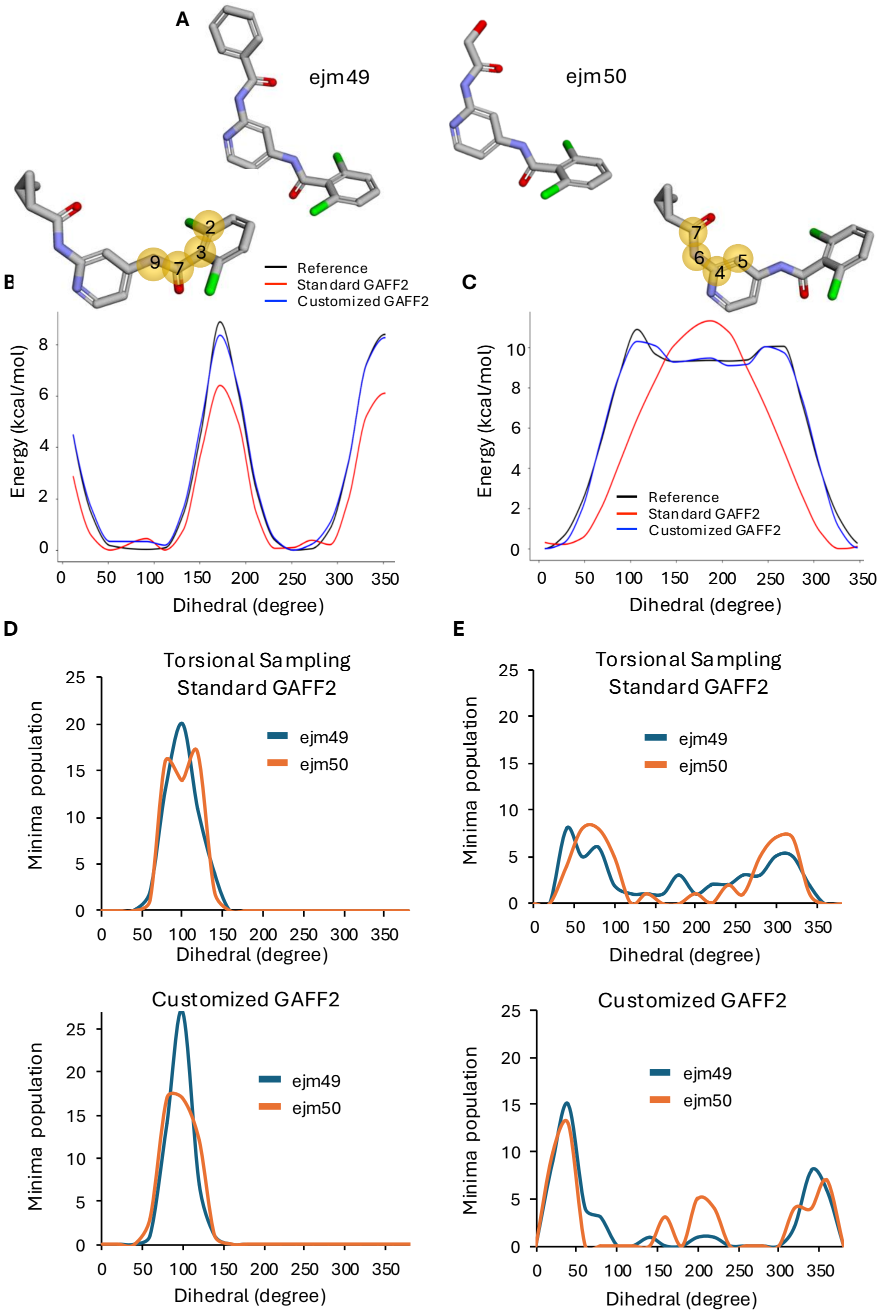

Figure S3: Comparative torsional sampling analysis between standard GAFF2 and customized GAFF2 for the ejm49-ejm50 ligand transformation (TYK2 system) in aqueous solution, an example where customized GAFF2 does not improve RBFE accuracy. (**A**) Chemical structures of ligands ejm49 and ejm50 show structural variations. (**B-C**) Reference torsional energy profiles (black) and corresponding profiles generated with standard GAFF2 (red) and customized GAFF2 (blue) for torsions 2-3-7-9 (**B**) and 7-6-4-5 (**C**). Insets show the re-parametrized ligand structure indicating the torsions analyzed. (**D-E**) Torsional sampling distributions of torsions 2-3-7-9 (**D**) and 7-6-4-5 (**E**) comparing standard GAFF2 (top panel) and customized GAFF2 (bottom panel) for ligands ejm49 and ejm50, show the population distribution across different PES minima. All panels report the dihedral angle scale in degrees (X-axis) using the absolute torsion values, with the minimum located at its corresponding dihedral angle.

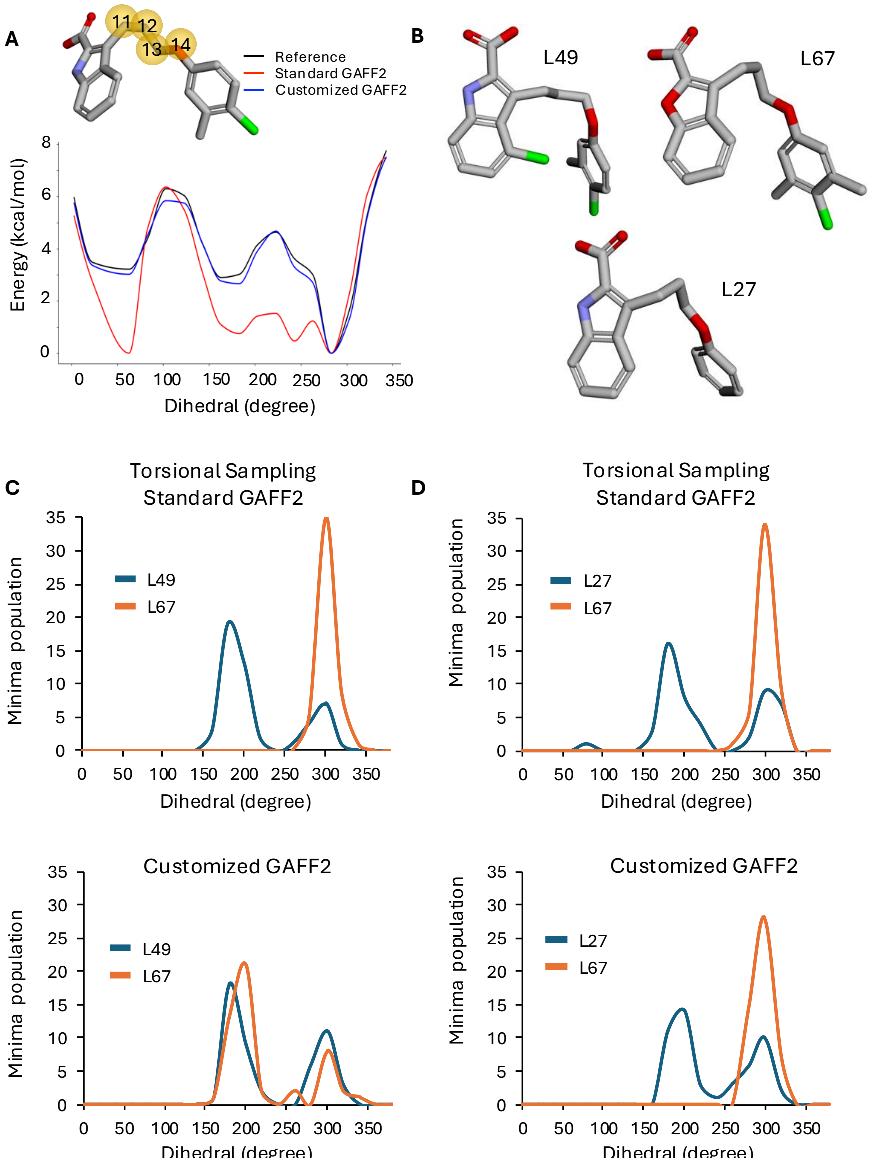

Figure S4: Comparative torsional sampling analysis between standard GAFF2 and customized GAFF2 for two ligand transformations in the MCL1 system in aqueous solution, illustrating a successful and an unsuccessful example of RBFE improvement. (**A**) Chemical structures of ligands L49, L67, and L27 show structural variations. (**B**) Reference torsional energy profile (black) and corresponding profiles generated with standard GAFF2 (red) and customized GAFF2 (blue) for torsion 11-12-13-14. Insets depict the re-parametrized ligand structure, highlighting the analyzed torsion. (**C**) Torsional sampling distributions for transformation L49–L67 (improved RBFE accuracy), comparing standard GAFF2 (top panel) and customized GAFF2 (bottom panel). (**D**) Torsional sampling distributions for transformation L67–L27 (no improvement in RBFE accuracy), comparing standard GAFF2 (top panel) and customized GAFF2 (bottom panel). All panels report the dihedral angle scale in degrees (X-axis) using the absolute torsion values, with the minimum located at its corresponding dihedral angle.

References

(1) Karwounopoulos, J.; Bieniek, M.; Wu, Z.; Baskerville, A. L.; König, G.; Cossins, B. P.; Wood, G. P. F. Evaluation of Machine Learning/Molecular Mechanics End-State Corrections with Mechanical Embedding to Calculate Relative Protein–Ligand Binding Free Energies. *J. Chem. Theory Comput.* **2025**, *21*, 967–977, PMID: 39753520.

(2) Wang, L. et al. Accurate and Reliable Prediction of Relative Ligand Binding Potency in Prospective Drug Discovery by Way of a Modern Free-Energy Calculation Protocol and Force Field. *J. Am. Chem. Soc.* **2015**, *137*, 2695–2703.

(3) Ganguly, A.; Tsai, H.-C.; Fernández-Pendás, M.; Lee, T.-S.; Giese, T. J.; York, D. M. AMBER Drug Discovery Boost Tools: Automated Workflow for Production Free-Energy Simulation Setup and Analysis (ProFESSA). *J. Chem. Inf. Model.* **2022**, *62*, 6069–6083.

(4) Tian, C.; Kasavajhala, K.; Belfon, K. A. A.; Raguette, L.; Huang, H.; Migues, A. N.; Bickel, J.; Wang, Y.; Pincay, J.; Wu, Q.; Simmerling, C. ff19SB: Amino-Acid-Specific Protein Backbone Parameters Trained against Quantum Mechanics Energy Surfaces in Solution. *J. Chem. Theory Comput.* **2020**, *16*, 528–552.

(5) He, X.; Man, V. H.; Yang, W.; Lee, T.-S.; Wang, J. A fast and high-quality charge model for the next generation general AMBER force field. *J. Chem. Phys.* **2020**, *153*, 114502.

(6) Horn, H. W.; Swope, W. C.; Pitera, J. W.; Madura, J. D.; Dick, T. J.; Hura, G. L.; Head-Gordon, T. Development of an improved four-site water model for biomolecular simulations: TIP4P-Ew. *J. Chem. Phys.* **2004**, *120*, 9665–9678.

(7) Lee, T.-S.; Lin, Z.; Allen, B. K.; Lin, C.; Radak, B. K.; Tao, Y.; Tsai, H.-C.; Sherman, W.; York, D. M. Improved Alchemical Free Energy Calculations with Optimized Smoothstep Softcore Potentials. *J. Chem. Theory Comput.* **2020**, *16*, 5512–5525.

(8) Darden, T.; York, D.; Pedersen, L. Particle mesh Ewald: An N·log(N) method for Ewald sums in large systems. *J. Chem. Phys.* **1993**, *98*, 10089–10092.

(9) Essmann, U.; Perera, L.; Berkowitz, M. L.; Darden, T.; Lee, H.; Pedersen, L. G. A smooth particle mesh Ewald method. *J. Chem. Phys.* **1995**, *103*, 8577–8593.

(10) Horton, J. T.; Boothroyd, S.; Wagner, J.; Mitchell, J. A.; Gokey, T.; Dotson, D. L.; Behara, P. K.; Ramaswamy, V. K.; Mackey, M.; Chodera, J. D.; Anwar, J.; Mobley, D. L.; Cole, D. J. Open Force Field BespokeFit: Automating Bespoke Torsion Parametrization at Scale. *J. Chem. Inf. Model.* **2022**, *62*, 5622–5633.

(11) Song, L. F.; Lee, T.-S.; Zhu, C.; York, D. M.; Merz, K. M., Jr. Using AMBER18 for Relative Free Energy Calculations. *J. Chem. Inf. Model.* **2019**, *59*, 3128–3135.

(12) Hahn, D. F.; Bayly, C. I.; Boby, M. L.; Macdonald, H. E. B.; Chodera, J. D.; Gapsys, V.; Mey, A. S.; Mobley, D. L.; Benito, L. P.; Schindler, C. E.; others Best practices for constructing, preparing, and evaluating protein-ligand binding affinity benchmarks [article v1. 0]. *Living J. Comput. Mol. Sci.* **2022**, *4*, 1497.
