# Supplementary material for: Automated Force Field Developer and Optimizer Platform: Torsion Reparameterization
Source: J Chem Inf Model. 2026 Mar 9;66(6):3206–19. doi: 10.1021/acs.jcim.6c00528 (PMC13014461; doi:10.1021/acs.jcim.6c00528)
Supplement: Supplementary file 2 [file ci6c00528_si_002.zip › input_files/AFFDO-runs/jmc28_f1-MS/resources/Instructions.docx]

**
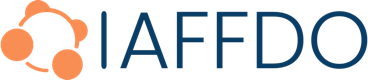
An Automated Force Field Developer and Optimizer.**

**Product Version**: AFFDO-24.09

**AFFDO** is designed to provide customized torsion parameters for small drug-like molecules, significantly enhancing the accuracy of binding free energy predictions. After completing a run with AFFDO, users receive a comprehensive package containing the input files, log files, and the final outputs needed for further computational analysis. The key deliverable from this package is the final *parmed* input file (.in), containing the newly optimized torsion parameters ready to update the Generalized Amber Force Field (GAFF) topology file employed during your molecular dynamics’ simulations.

# **Project folder structure**

1. **ParmEd input file (.in):**
   - This file contains the newly optimized torsion parameters and is the primary output of the AFFDO workflow. Users should utilize this file in conjunction with the AMBER molecular dynamics package to update their simulation topology files, ensuring that the improved torsion parameters are incorporated into subsequent simulations.
2. **AFFDO output file (.log):**
   - The log file captures the entire process of the AFFDO workflow, providing insights into how the torsion parameters were optimized and can be used to verify that the workflow completed as expected.
3. **Complementary script (update_topology.py):**
   - This script uses ParmEd to apply updated torsion parameters to the standard GAFF/GAFF2 topology. In dual‑topology mode, it filters and updates only those torsions common to both ligands.
4. **Supporting files and folders:**
   - **Mol file (.mol):** The standardized MOL-format file containing the ligand’s coordinates and atom labels as processed by AFFDO.
   - **Data JSON (.json):** Contains metadata and settings used in the AFFDO run.
   - **Input file folder:** The original upload (PDB/MOL2/MOL) is preserved in this folder.
   - **Standard GAFF topology folder:** Contains the unmodified GAFF/GAFF2 topology files for the original ligand (.prmtop, .rst7, .mol2, .frcmod) for user reference.
   - **Results folder:** Contains intermediate files generated during the AFFDO workflow, such as conformers generation, centroid_optimization, torsional_scan, paramopt_works, etc. These files can be valuable for users interested in the detailed steps of the parameter optimization process.

If the fragmentation algorithm was executed:

- **Fragments image** (.png): A visual representation of the fragments analyzed and parametrized.
- **Fragment results folder:** project_f1, project_f2, etc.

5.**Topology‑Only Mode (optional)**

- If you invoked AFFDO with the “Topology‑Only” mode, AFFDO will stop as soon as it builds the standard GAFF/GAFF2 topology. No reparameterization related files will be included.

6.**Fragmentation‑Only Mode (optional)**

- If you invoked AFFDO with the “Fragmentation‑Only” mode, AFFDO fragments the ligand and then immediately exits. No topology or torsion reparameterization is performed.

# **Updating the GAFF topology file through ParmEd**

Once AFFDO has generated your ParmEd update file (e.g. params_update.in), you can inject those torsion parameters into your GAFF/GAFF2 topology using our helper script: **update_topology.py.** This tool automates all the ParmEd calls. Under dual‑topology mode, it keeps only those torsion modifications that appear in both ligand states. This ensures consistent torsion updates across end‑states, ideal for alchemical free‑energy simulations.

**Usage:** To run the script, use the following command:

- python update_topology.py -p *TOPOLOGY.parm7* -i *params_update.in* [--dual-topology]

**Required arguments:**

- -p | --topology: Path to your Amber GAFF/GAFF2 topology file (.parm7).
- -i | --update-in: AFFDO-generated ParmEd torsion update script (.in file).

**Optional arguments:**

- --dual-topology: Apply updates only to torsions common to two ligands (residues 1 and 2).
- -h | --help: Display detailed help and examples.

**Example:**

# Single topology (standard usage):

- python update_topology.py -p my_system.parm7 -i params_update.in

# Dual topology (common torsions only):

- python update_topology.py -p my_system.parm7 -i params_update.in --dual-topology

This generates a new topology file named new.prmtop incorporating the optimized torsion parameters.

**Notes:**

- Ensure Python 3 and ParmEd are installed and accessible from your command line.
- Use python update_topology.py -h to display this help message at any time.

**Tips for Safe Topology Updates**

When you move from AFFDO’s torsion re‑parameterization into your MD or RBFE workflows, keep these in mind:

1. **Use identical ligand coordinates**: The exact same .mol / .pdb / .mol2 you gave AFFDO must be the one your MD engine reads. Any atom‑order or numbering mismatch will cause ParmEd to silently skip torsion updates, so double‑check file consistency.
2. **Inspect ParmEd logs for errors**: After running update_topology.py (or your own ParmEd invocation), always glance at the generated log (parmed_update.log) or console output for warnings. That ensures you don’t inadvertently launch production runs with default GAFF/GAFF2 torsions.
3. **(Optional) Dual‑topology for RBFE**: If you’re doing a two‑end‑state free‑energy transform, we *recommend* updating only the torsions shared by both ligands. Our --dual-topology flag in update_topology.py makes this easy, but you’re free to use ParmEd directly if you prefer a different strategy.

Those few checks will give you confidence that your AFFDO‑refitted torsions made it into your final topology.

**Reference**

For additional details, please refer to reference below or consult the [AFFDO documentation](https://attmos.github.io/AFFDOWS/index.html).

If you use the AFFDO web service, please cite the following reference:

*Blanco-Gonzalez, A.; Betancourt, W.; Snyder, R.; Zhang, S.; Giese, T. J.; Goetz, A. W.; Merz, K. M., Jr.; York, D. M.; Aktulga, H. M.; Manathunga, M. Automated Force Field Developer and Optimizer Platform: Torsion Reparameterization. ChemRxiv 2024, doi:10.26434/chemrxiv-2024-lcnx1.*

**Disclaimer**

Thank you for using AFFDO web service. Please be advised of the following:

1. **Active Development:** This server is part of an active development project. While we strive to provide accurate and reliable results, the technology and underlying methods are continuously evolving. Consequently, there may be occasional updates and changes that could affect the output.
2. **Accuracy of Results:** The developers and hosting institution (San Diego Supercomputer Center) take no responsibility for the accuracy, reliability, or validity of the results generated by this server. Users are advised to critically evaluate the results and use them at their own risk.
3. **Performance:** The performance of calculations may vary depending on the current load and availability of resources. Hardware support is provided by the San Diego Supercomputer Center, and all calculations are submitted to a queue system. Therefore, runtimes are subject to fluctuations based on the queue status and system usage.
4. **No Liability:** By using this server, you agree that the developers and hosting institution will not be held liable for any direct, indirect, incidental, or consequential damages arising from the use of the results generated. This includes, but is not limited to, any issues related to data integrity, scientific conclusions, or any other use cases.
5. **Data Usage:** By using this server, you acknowledge and agree that your input data may be collected and used to improve the methodology and performance of our technology.

# **Contact & Feedback**

For any comments, queries, or issues, you can reach out to us via:

- **Email:** [affdo@attmosdiscovery.com](mailto:affdo@attmosdiscovery.com)

- **GitHub:** [AFFDOWS Issues or Discussions](https://github.com/ATTMOS/AFFDOWS)

We appreciate your feedback as it helps improve the tool and the service we offer.
