# Supplementary material for: Automated Force Field Developer and Optimizer Platform: Torsion Reparameterization
Source: J Chem Inf Model. 2026 Mar 9;66(6):3206–19. doi: 10.1021/acs.jcim.6c00528 (PMC13014461; doi:10.1021/acs.jcim.6c00528)
Supplement: Supplementary file 2 [file ci6c00528_si_002.zip › input_files/AFFDO-runs/mcl1_35_f1-MS/resources/Instructions.docx]

**
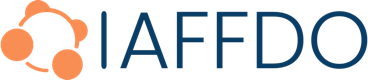
An Automated Force Field Developer and Optimizer.**

**Product Version**: AFFDO-24.09

**AFFDO** is designed to provide customized torsion parameters for small drug-like molecules, significantly enhancing the accuracy of binding free energy predictions. After completing a run with AFFDO, users receive a comprehensive package containing the input files, log files, and the final outputs needed for further computational analysis. The key deliverable from this package is the final *parmed* input file (.in), containing the newly optimized torsion parameters ready to update the Generalized Amber Force Field (GAFF) topology file employed during your molecular dynamics’ simulations.

**Workflow Modes**

- **Parametrization Mode (default):** Full workflow including fragmentation (if applicable), QM/MM scans, parameter optimization, and generation of updated torsion parameters.
- **Topology-Only Mode (optional):** AFFDO stops immediately after building base GAFF/GAFF2 topology. No reparameterization files (no workflow_files/, stats_summary/, etc.).
- **Fragmentation-Only Mode (optional):** AFFDO generates fragments and exits. No topology or parameter fitting is performed.

**Folder Structure Description**

The AFFDO output directory is organized into logical components that separate final deliverables, workflow metadata, diagnostic plots, and fragment-level computations. Below is a description of the main folders and the key files users typically interact with.

- **Project (.log):** The full AFFDO workflow log, recording every step of the parametrization process. Useful for verifying successful execution and diagnosing potential issues.
- **Mol file (.mol):** The standardized MOL-format file containing the ligand’s coordinates and atom labels as processed by AFFDO.
- **Data JSON (.json):** Contains metadata and settings used in the AFFDO run.
- **Fitting Report (.html):** Interactive HTML report. This is the primary visual summary of the AFFDO run.
- **Topology (.zip):** Archive containing unmodified GAFF/GAFF2 topology files created before reparameterization (prmtop, rst7, mol2, frcmod). These are provided for reference and optional comparison.
- **Workflow Files Folder**
  - **ParmEd input file (.in):** The most important final output of AFFDO. Contains the optimized torsion parameters in ParmEd format. This file must be passed to update_topology.py or your own ParmEd script to update the GAFF/GAFF2 topology. In dual‑topology mode, it filters and updates only those torsions common to both ligands.
  - **Update Topology file (.frcmod):** GAFF-style .frcmod file version of the optimized torsions (optional alternate format).
  - **Run configuration (.txt):** Readable record of all settings used in the workflow.
- **Stats Summary Folder**
  - **Summary dihedral plots (.png):** Energy comparison plots: Reference vs GAFF2 vs AFFDO for each optimized torsion.
  - **Fitting Summary (.txt):** Human-readable summary of RMSE values and improvements.
  - **Fitting Report (.csv):** Machine-readable metrics for all torsions.
- **Input File Folder**
  - Input_file: The original user-uploaded structure (PDB/MOL2/MOL). Preserved for reference and reproducibility.
- **Resources Folder**
  - Contains static assets required for the interactive HTML report (logos, JS libraries, help files). Users do not need to modify these.
- **Fragment Folders (_f1.zip, _f2.zip, …)**
  - A compressed version of the complete fragment folder for easy download
  - Only created when fragmentation is active. Each fragment folder contains the fragment structure and log, local and parent-mapped torsion parameter files, and per-fragment metadata (data.json)
  - **Results subfolder:** Contain intermediate QM/MM, clustering, scan, and optimization data. These assist debugging but are not needed for normal usage.

**Folder Structure Diagram**

project_root/

│

├── <project>.log

├── <project>.mol

├── data.json

├── fitting_report.html

├── topology.zip

│

├── workflow_files/

│ ├── update_params_<project>.in

│ ├── update_params_<project>.frcmod
 │ ├── run_configuration.txt

│ └── <project>.log

│

├── stats_summary/

│ ├── <project>_fragments.png

│ ├── dh_X-X-X-X_<fragment>.png

│ ├── fitting_summary.txt

│ └── fitting_report.csv

│

├── input_file/

│ └── <input_filename>

│

├── resources/

│ ├── Attmos_logo.png

│ ├── logo.txt

│ ├── Instructions.docx

│ └── js/

│

├── <project>_f1.zip

│ ├── <project>_f1.log

│ ├── <project>_f1.mol

│ ├── data.json

│ ├── update_params_<project>_f1.in

│ ├── update_params_mapped_<project>_f1.in

│ └── results/

├── <project>_f2.zip

└── <project>_f3.zip

# **Updating the GAFF topology file through ParmEd**

Once AFFDO has generated your ParmEd update file (e.g. params_update.in), you can inject those torsion parameters into your GAFF/GAFF2 topology using our helper script: **update_topology.py.** This tool automates all the ParmEd calls. Under dual‑topology mode, it keeps only those torsion modifications that appear in both ligand states. This ensures consistent torsion updates across end‑states, ideal for alchemical free‑energy simulations.

**Usage:** To run the script, use the following command:

- python update_topology.py -p *TOPOLOGY.parm7* -i *params_update.in* [--dual-topology]

**Required arguments:**

- -p | --topology: Path to your Amber GAFF/GAFF2 topology file (.parm7).
- -i | --update-in: AFFDO-generated ParmEd torsion update script (.in file).

**Optional arguments:**

- --dual-topology: Apply updates only to torsions common to two ligands (residues 1 and 2).
- -h | --help: Display detailed help and examples.

**Example:**

# Single topology (standard usage):

- python update_topology.py -p my_system.parm7 -i params_update.in

# Dual topology (common torsions only):

- python update_topology.py -p my_system.parm7 -i params_update.in --dual-topology

This generates a new topology file named new.prmtop incorporating the optimized torsion parameters.

**Notes:**

- Ensure Python 3 and ParmEd are installed and accessible from your command line.
- Use python update_topology.py -h to display this help message at any time.

**Tips for Safe Topology Updates**

When you move from AFFDO’s torsion re‑parameterization into your MD or RBFE workflows, keep these in mind:

1. **Use identical ligand coordinates**: The exact same .mol / .pdb / .mol2 you gave AFFDO must be the one your MD engine reads. Any atom‑order or numbering mismatch will cause ParmEd to silently skip torsion updates, so double‑check file consistency.
2. **Inspect ParmEd logs for errors**: After running update_topology.py (or your own ParmEd invocation), always glance at the generated log (parmed_update.log) or console output for warnings. That ensures you don’t inadvertently launch production runs with default GAFF/GAFF2 torsions.
3. **(Optional) Dual‑topology for RBFE**: If you’re doing a two‑end‑state free‑energy transform, we *recommend* updating only the torsions shared by both ligands. Our --dual-topology flag in update_topology.py makes this easy, but you’re free to use ParmEd directly if you prefer a different strategy.

Those few checks will give you confidence that your AFFDO‑refitted torsions made it into your final topology.

**Reference**

For additional details, please refer to reference below or consult the [AFFDO documentation](https://attmos.github.io/AFFDOWS/index.html).

If you use the AFFDO web service, please cite the following reference:

*Blanco-Gonzalez, A.; Betancourt, W.; Snyder, R.; Zhang, S.; Giese, T. J.; Piskulich, Z. A.; Goetz, A. W.; Merz, K. M., Jr.; York, D. M.; Aktulga, H. M.; Manathunga, M. Automated Force Field Developer and Optimizer Platform: Torsion Reparameterization. ChemRxiv 2024, doi:10.26434/chemrxiv-2024-lcnx1.*

**Disclaimer**

Thank you for using AFFDO web service. Please be advised of the following:

1. **Active Development:** This server is part of an active development project. While we strive to provide accurate and reliable results, the technology and underlying methods are continuously evolving. Consequently, there may be occasional updates and changes that could affect the output.
2. **Accuracy of Results:** The developers and hosting institution (San Diego Supercomputer Center) take no responsibility for the accuracy, reliability, or validity of the results generated by this server. Users are advised to critically evaluate the results and use them at their own risk.
3. **Performance:** The performance of calculations may vary depending on the current load and availability of resources. Hardware support is provided by the San Diego Supercomputer Center, and all calculations are submitted to a queue system. Therefore, runtimes are subject to fluctuations based on the queue status and system usage.
4. **No Liability:** By using this server, you agree that the developers and hosting institution will not be held liable for any direct, indirect, incidental, or consequential damages arising from the use of the results generated. This includes, but is not limited to, any issues related to data integrity, scientific conclusions, or any other use cases.
5. **Data Usage:** By using this server, you acknowledge and agree that your input data may be collected and used to improve the methodology and performance of our technology.

# **Contact & Feedback**

For any comments, queries, or issues, you can reach out to us via:

- **Email:** [affdo@attmosdiscovery.com](mailto:affdo@attmosdiscovery.com)

- **GitHub:** [AFFDOWS Issues or Discussions](https://github.com/ATTMOS/AFFDOWS)

We appreciate your feedback as it helps improve the tool and the service we offer.
