# Supplementary figures and images for: Automated Force Field Developer and Optimizer Platform: Torsion Reparameterization
Source: J Chem Inf Model. 2026 Mar 9;66(6):3206–19. doi: 10.1021/acs.jcim.6c00528 (PMC13014461; doi:10.1021/acs.jcim.6c00528)

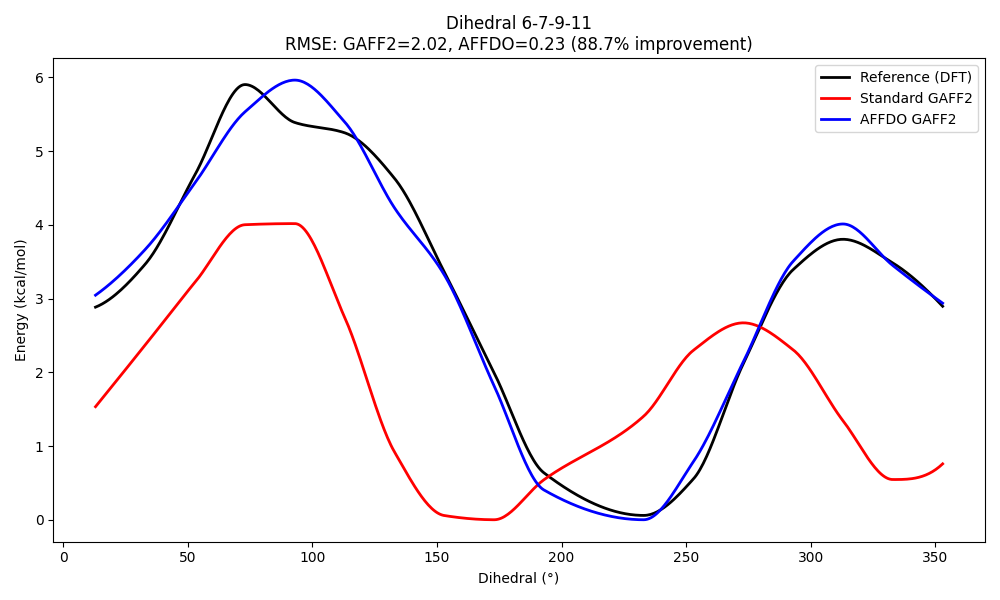

Supplement: Supplementary file 2 [file ci6c00528_si_002.zip › input_files/AFFDO-runs/jmc28_f1-MS/stats_summary/confs_999-999_dh_6-7-9-11.png]

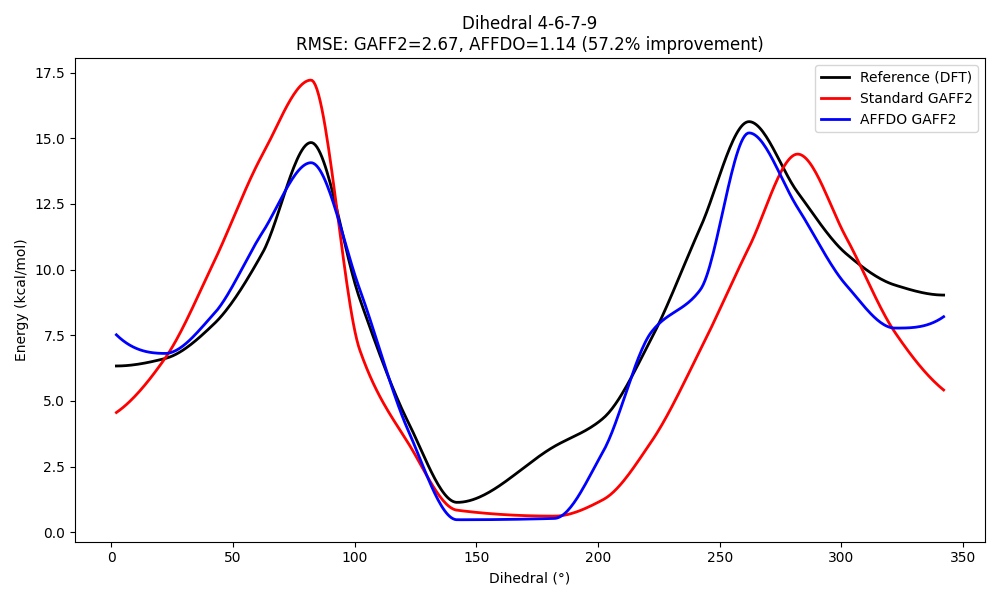

Supplement: Supplementary file 2 [file ci6c00528_si_002.zip › input_files/AFFDO-runs/jmc28_f1-MS/stats_summary/confs_999-999_dh_4-6-7-9.png]

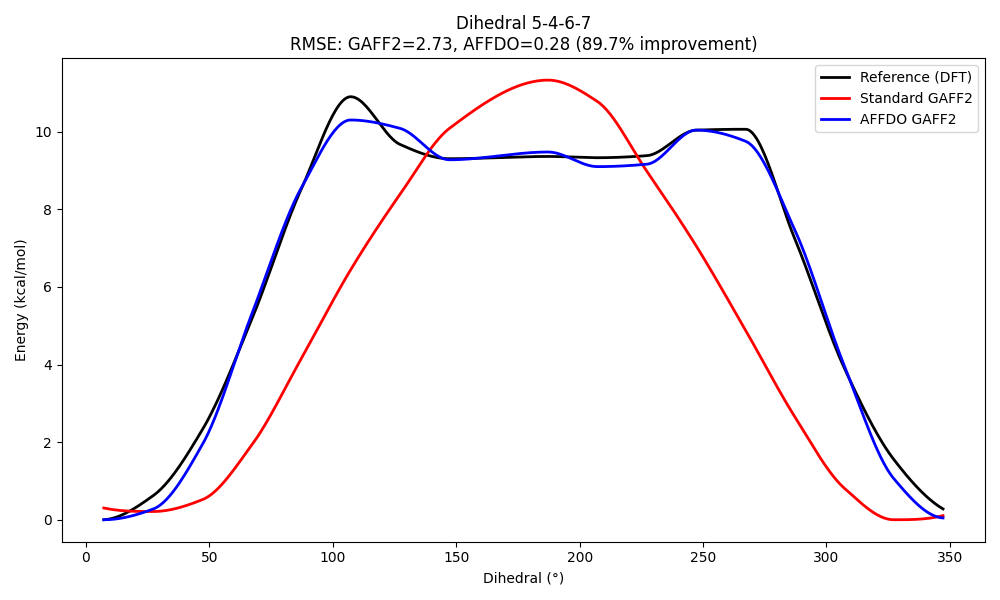

Supplement: Supplementary file 2 [file ci6c00528_si_002.zip › input_files/AFFDO-runs/jmc28_f1-MS/stats_summary/confs_999-999_dh_5-4-6-7.png]

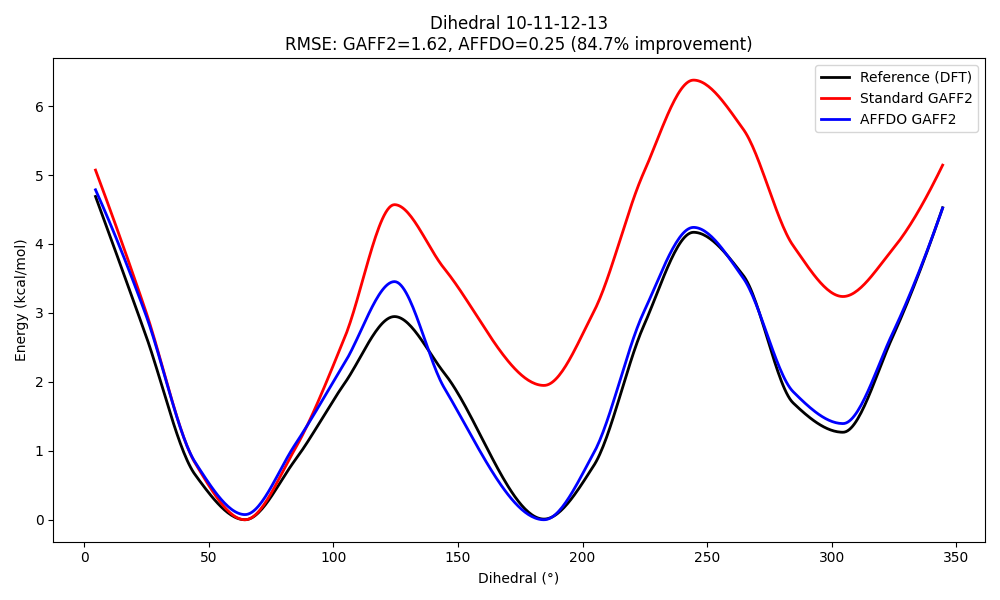

Supplement: Supplementary file 2 [file ci6c00528_si_002.zip › input_files/AFFDO-runs/mcl1_35_f1-MS/stats_summary/confs_999-999_dh_10-11-12-13.png]

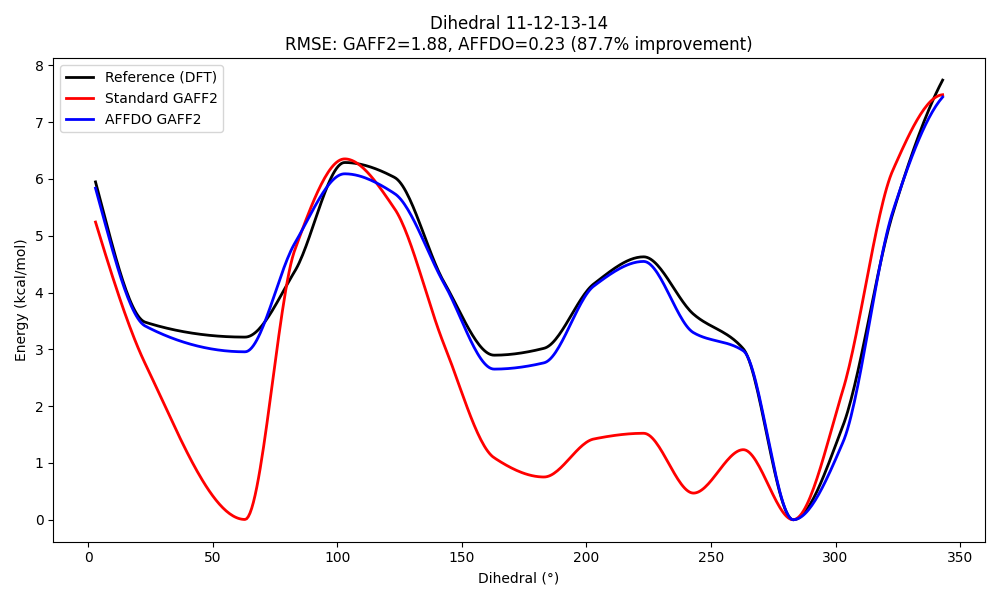

Supplement: Supplementary file 2 [file ci6c00528_si_002.zip › input_files/AFFDO-runs/mcl1_35_f1-MS/stats_summary/confs_999-999_dh_11-12-13-14.png]

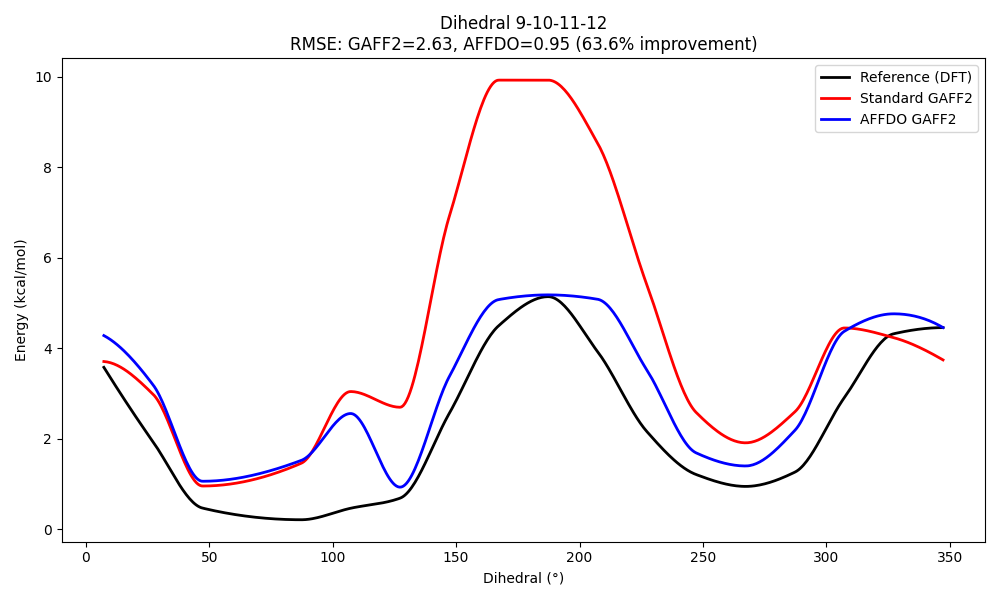

Supplement: Supplementary file 2 [file ci6c00528_si_002.zip › input_files/AFFDO-runs/mcl1_35_f1-MS/stats_summary/confs_999-999_dh_9-10-11-12.png]

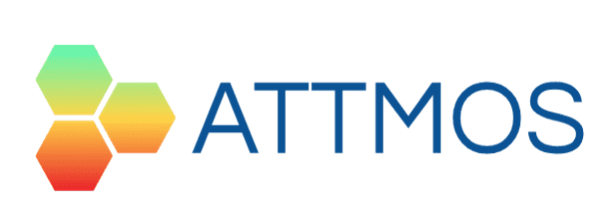

Supplement: Supplementary file 2 [file ci6c00528_si_002.zip › input_files/AFFDO-runs/mcl1_35_f1-MS/resources/Attmos_logo.png]

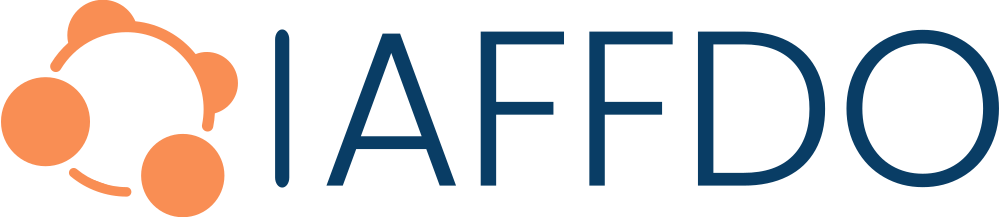

Supplement: Supplementary file 2 [file ci6c00528_si_002.zip › input_files/AFFDO-runs/mcl1_35_f1-MS/resources/logo-no-background.png]

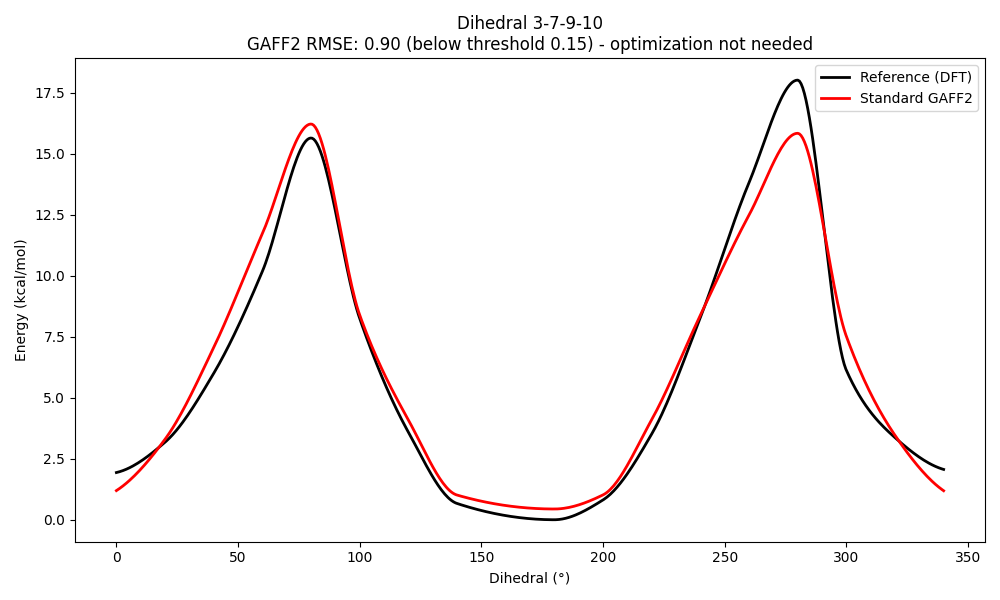

Supplement: Supplementary file 2 [file ci6c00528_si_002.zip › input_files/AFFDO-runs/jmc28_f2-MS/stats_summary/confs_999-999_dh_3-7-9-10.png]

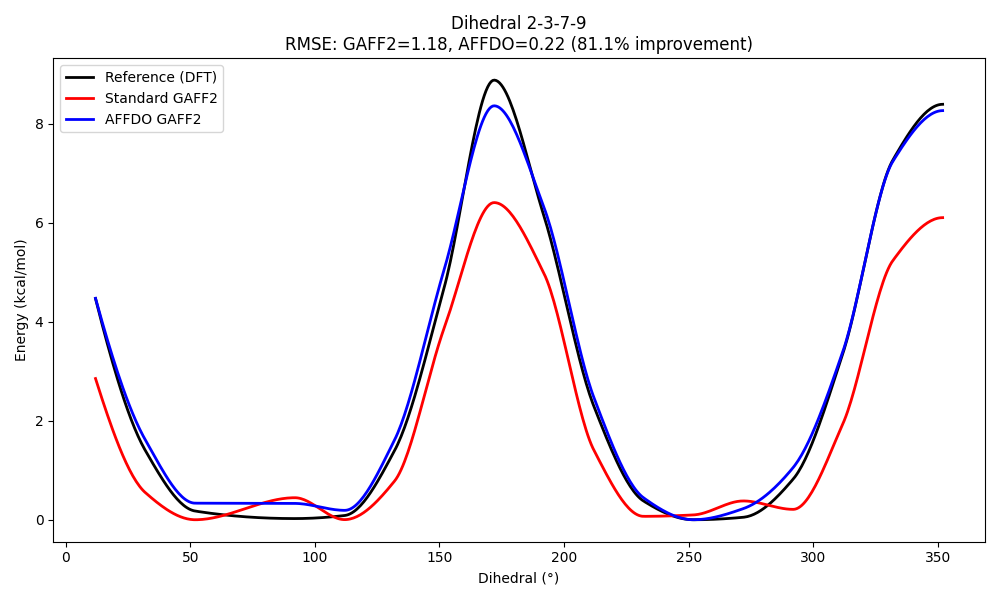

Supplement: Supplementary file 2 [file ci6c00528_si_002.zip › input_files/AFFDO-runs/jmc28_f2-MS/stats_summary/confs_999-999_dh_2-3-7-9.png]

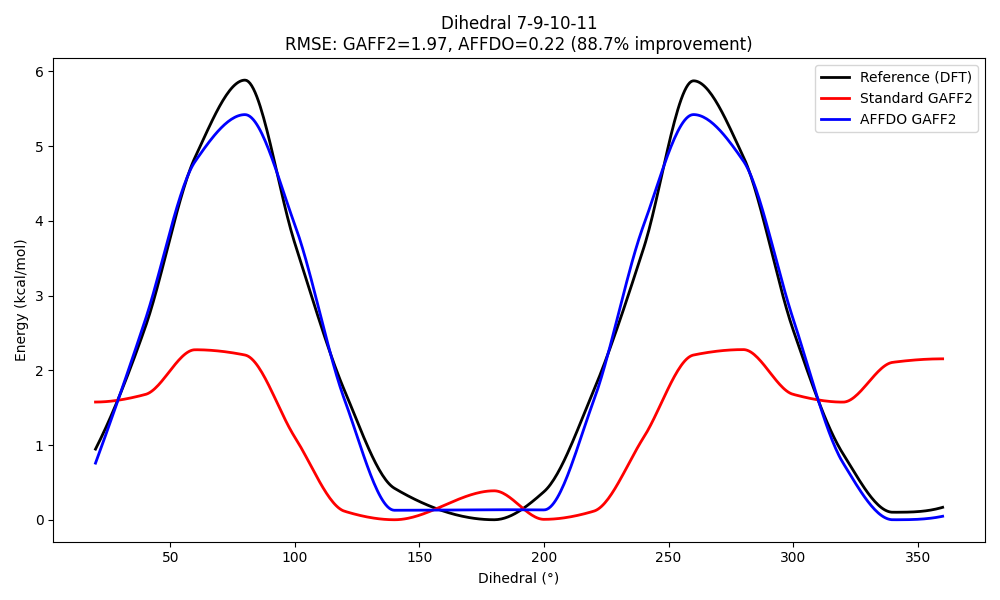

Supplement: Supplementary file 2 [file ci6c00528_si_002.zip › input_files/AFFDO-runs/jmc28_f2-MS/stats_summary/confs_999-999_dh_7-9-10-11.png]

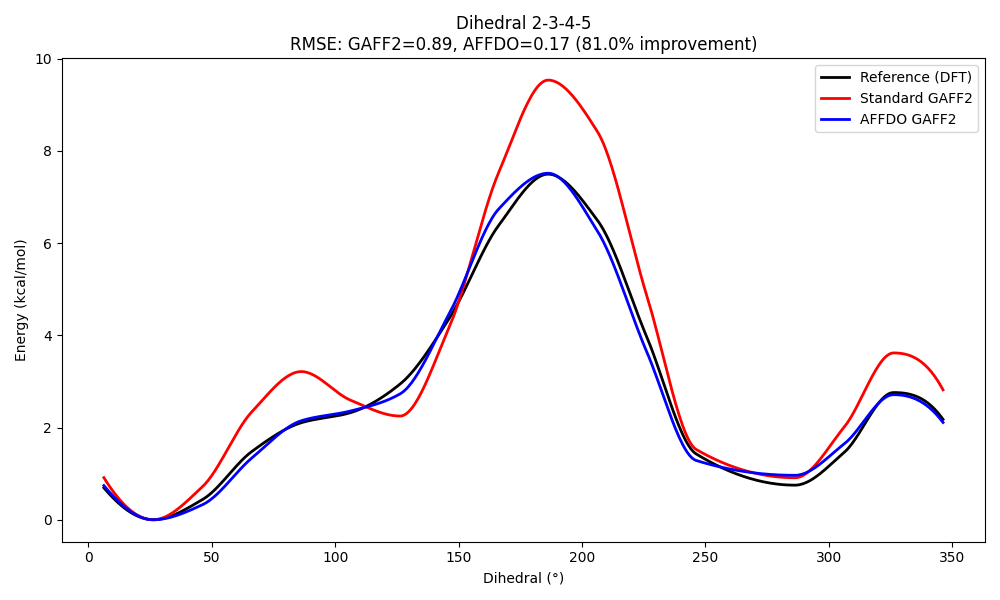

Supplement: Supplementary file 2 [file ci6c00528_si_002.zip › input_files/AFFDO-runs/mcl1_35_f2-MS/stats_summary/confs_999-999_dh_2-3-4-5.png]

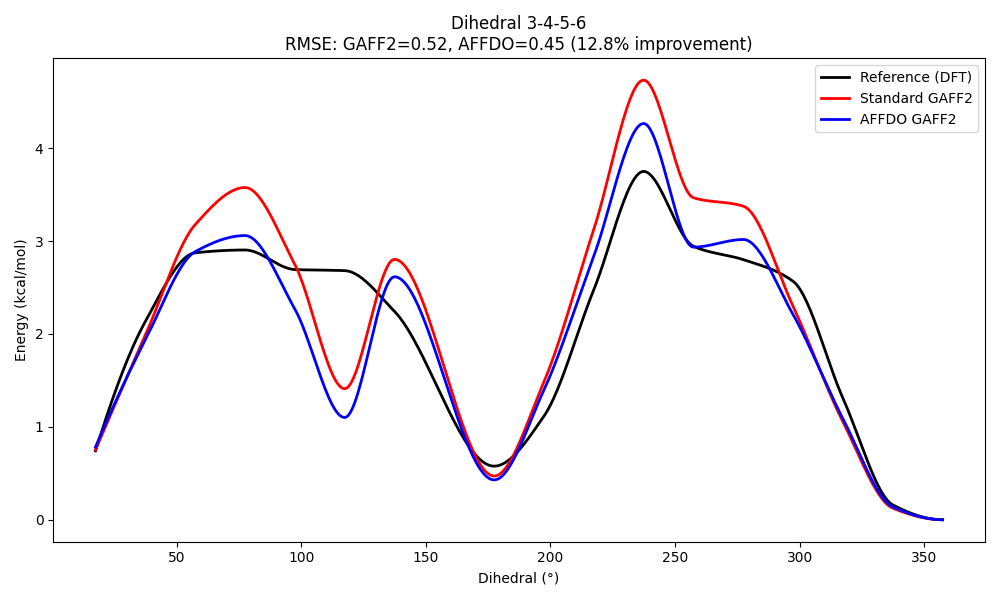

Supplement: Supplementary file 2 [file ci6c00528_si_002.zip › input_files/AFFDO-runs/mcl1_35_f2-MS/stats_summary/confs_999-999_dh_3-4-5-6.png]

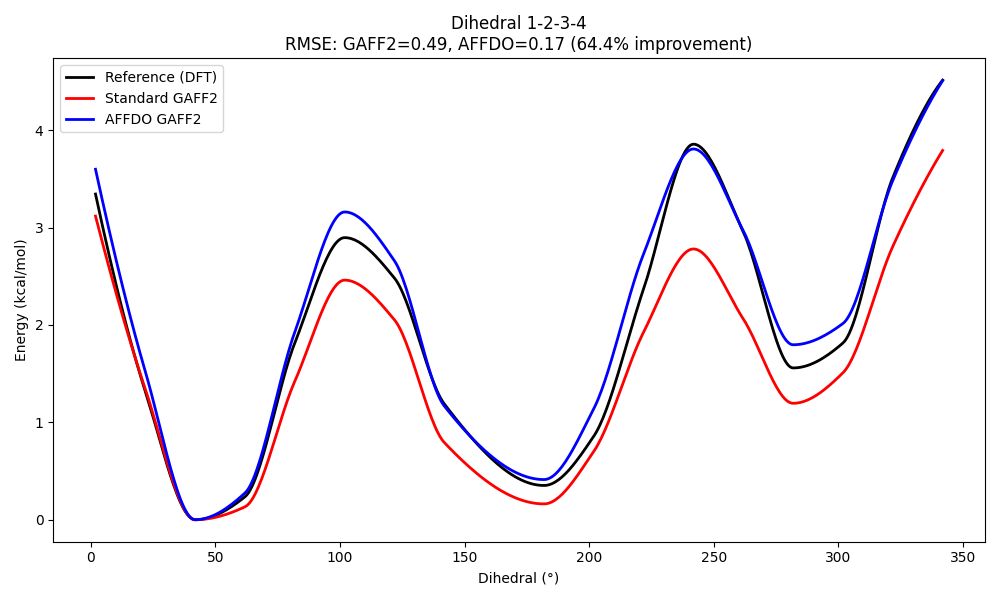

Supplement: Supplementary file 2 [file ci6c00528_si_002.zip › input_files/AFFDO-runs/mcl1_35_f2-MS/stats_summary/confs_999-999_dh_1-2-3-4.png]

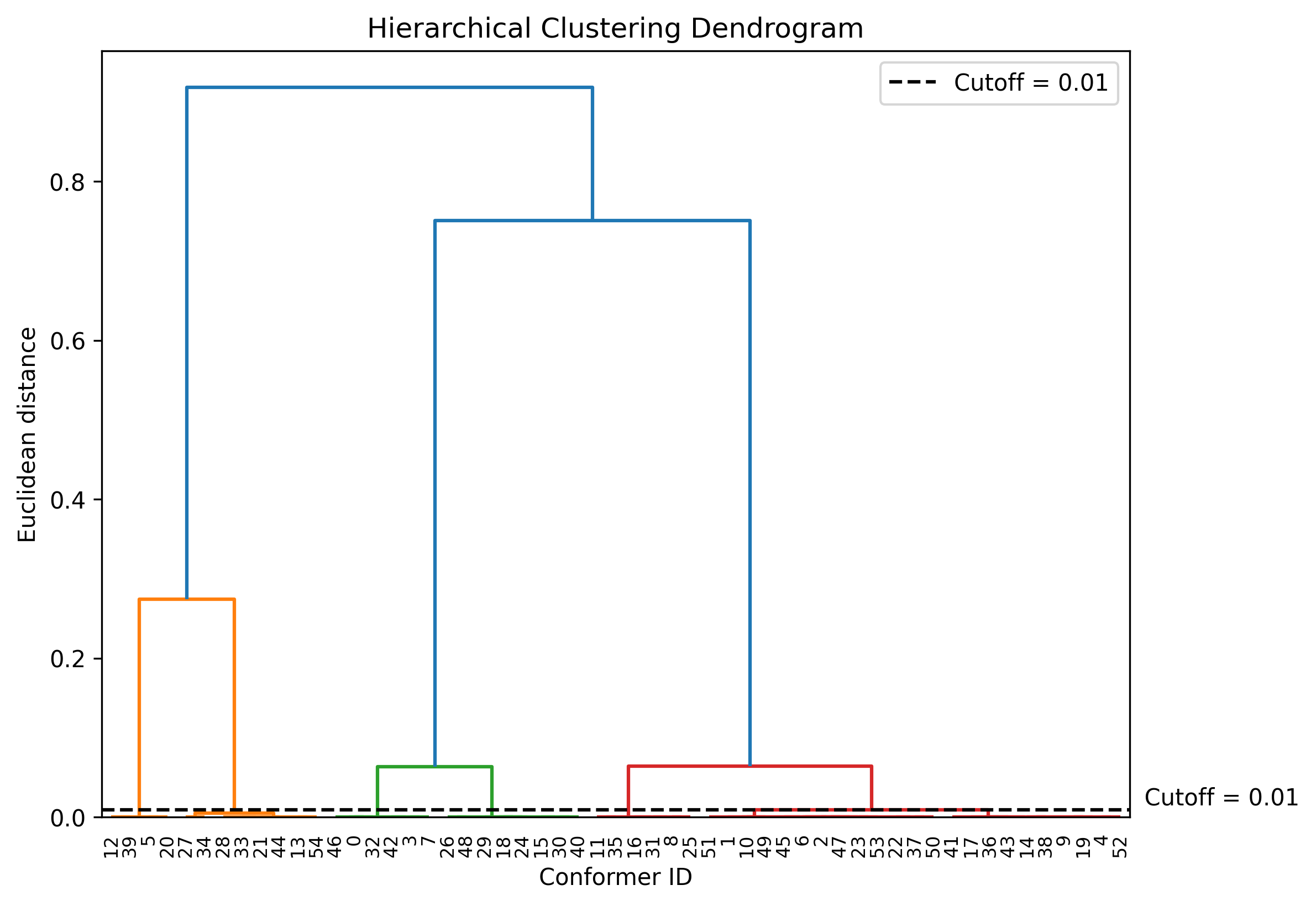

Supplement: Supplementary file 2 [file ci6c00528_si_002.zip › input_files/AFFDO-runs/jmc28_f1-MS/results/clusters/dendrogram.png]
